# TCMNPAS: a comprehensive analysis platform integrating network formulaology and network pharmacology for exploring traditional Chinese medicine

**DOI:** 10.1186/s13020-024-00924-y

**Published:** 2024-03-22

**Authors:** Yishu Liu, Xue Li, Chao Chen, Nan Ding, Peiyong Zheng, Xiaoyun Chen, Shiyu Ma, Ming Yang

**Affiliations:** 1 LongHua Hospital Shanghai University of Traditional Chinese Medicine, Shanghai, 200032 China; 2 Ruijin Hospital Affiliated to Shanghai Jiaotong University School of Medicine, Shanghai, 200025 China

**Keywords:** TCMNPAS, Network pharmacology analysis system, Traditional Chinese medicine, Molecular mechanism, Core formula mining, Molecular docking

## Abstract

The application of network formulaology and network pharmacology has significantly advanced the scientific understanding of traditional Chinese medicine (TCM) treatment mechanisms in disease. The field of herbal biology is experiencing a surge in data generation. However, researchers are encountering challenges due to the fragmented nature of the data and the reliance on programming tools for data analysis. We have developed TCMNPAS, a comprehensive analysis platform that integrates network formularology and network pharmacology. This platform is designed to investigate in-depth the compatibility characteristics of TCM formulas and their potential molecular mechanisms. TCMNPAS incorporates multiple resources and offers a range of functions designed for automated analysis implementation, including prescription mining, molecular docking, network pharmacology analysis, and visualization. These functions enable researchers to analyze and obtain core herbs and core formulas from herbal prescription data through prescription mining. Additionally, TCMNPAS facilitates virtual screening of active compounds in TCM and its formulas through batch molecular docking, allowing for the rapid construction and analysis of networks associated with “herb-compound-target-pathway” and disease targets. Built upon the integrated analysis concept of network formulaology and network pharmacology, TCMNPAS enables quick point-and-click completion of network-based association analysis, spanning from core formula mining from clinical data to the exploration of therapeutic targets for disease treatment. TCMNPAS serves as a powerful platform for uncovering the combinatorial rules and mechanism of TCM formulas holistically. We distribute TCMNPAS within an open-source R package at GitHub (https://github.com/yangpluszhu/tcmnpas), and the project is freely available at http://54.223.75.62:3838/.

**Supplementary Information:**

The online version contains supplementary material available at 10.1186/s13020-024-00924-y.

## Introduction

Traditional Chinese medicine (TCM) is a therapeutic approach that heavily relies on the application of TCM prescriptions. These prescriptions, formulated based on syndrome differentiation, play a significant role in TCM treatment. These prescriptions encompass a holistic approach to addressing various diseases and health conditions. To gain a comprehensive understanding of the principles of TCM treatment, it is essential to summarize the patterns of prescription composition and identify the active ingredients within these prescriptions. By conducting intricate analyses of these active ingredients, we can elucidate the potential molecular mechanisms underlying TCM’s efficacy in treating diseases. This process illuminates the complex interactions between TCM formulas and the human body.

The development of network formulaology (NF) and network pharmacology (NP) has emerged prominently in the field of traditional Chinese medicine (TCM) [1]. NF integrates holistic thinking patterns in TCM theories, constructing and analyzing interconnected networks to explore changes in efficacy resulting from different herb combinations in TCM formulas. Furthermore, NF investigates the relationship between the active ingredients of herbs in formulas and the biological molecular regulatory networks, delving deeper into the scientific meaning of the theory of herbal compatibility [2].

NP challenges the traditional paradigm of “one disease-one target-one drug” by exploring interactions between the drug and the body, mapping the drug-target-disease network on a biological level [3, 4]. In recent years, this approach has led to significant advancements in our understanding of drug action and protein–protein interactions [5, 6], consequently improving therapeutic strategies and the drug discovery process [7–11]. These developments have positioned NP as a transformative technology with the potential to bridge the gap between traditional and modern medicine, driving changes in methods for the rational design and optimization of drug discovery from herbal formulas [12, 13].

The framework and practice guide of network-based studies for understanding the mechanism of TCM formulas was presented [14]. However, the construction of a network relies on a diverse set of resources encompassing a wide range of TCM knowledge, biological processes, and a variety of computational algorithm tools. These resources are essential to ensure the successful integration of NF and NP into TCM research practices, ultimately advancing the study and application of TCM in modern healthcare.

Although extensive data sources are available, such as TCMSP [15], TCMID [16], TCMIP [17], ETCM [18] and BATMAN-TCM [19], effectively demonstrating the complicated process of TCM treating diseases remains challenging. Furthermore, integrating results from multiple databases can be time-consuming and demanding. To address these challenges, we have developed the TCM Network formulaology and Pharmacology Analysis System (TCMNPAS), a comprehensive platform that enables the analysis of “core formulas with core herbs and core targets” using a network approach. By integrating multiple databases, employing various algorithm techniques, and utilizing visualization analysis methods, TCMNPAS facilitates the analysis of TCM formulas and their active components, providing valuable insights into their therapeutic potential.

Recently, several platforms have been developed for TCM exploration. A summary of these platforms can be found in Additional file 1: Table S1. While these platforms share data retrieval capabilities, some like ETCM v2.0 [18] and BATMAN-TCM v2.0 [19] offer online analysis using a similarity-based computational framework to identify targets and support integrated system analysis, thus aiding in TCM-derived drug discovery and repurposing. However, they are not specifically designed for NF analysis. In contrast, TCMNPAS prioritizes the integration of network formularology and pharmacology, employing advanced statistical methods such as the binomial formula-target identification model [20–22], BK algorithm for prescription mining [23–25], network-based proximity measures [21, 22, 26], and molecular docking [27–31]. Our platform allows for customizable thresholds in data analysis and offers enhanced visualization tools, facilitating exploration of herb compatibility, reliable target evaluation, and in-depth analysis of the pharmacological mechanisms in TCM formulas. Additionally, TCMNPAS offers bilingual support in Chinese and English, enhancing accessibility to TCM research for a diverse range of domestic and international researchers.

TCMNPAS is a valuable tool for scholars seeking deeper insights into TCM formulas and their molecular mechanisms, thus contributing to the modernization and scientific understanding of TCM. TCMNPAS has been awarded the copyright by the National Copyright Administration of China (Certificate Registration Number: 2019SR1127090). We distribute TCMNPAS within an open-source R package at GitHub (https://github.com/yangpluszhu/tcmnpas), and the project is freely available at http://54.223.75.62:3838/.

## Materials and methods

TCMNPAS integrates multiple resources, including herbs, ingredients, and targets. Several TCM databases (TCMSP [15], HIT [32], and TCMID) have been incorporated into TCMNPAS. Moreover, TCMNPAS also incorporates compound-target data from STITCH [33], protein–protein interaction data from HIPPIE [34], as well as target-pathway data from KEGG [35] and Reactome [36]. This comprehensive integration allows for easy retrieval of multiple association data, such as herb-ingredient links, ingredient-target links, and herbal-ingredient-target links.

**Fig. 1:**
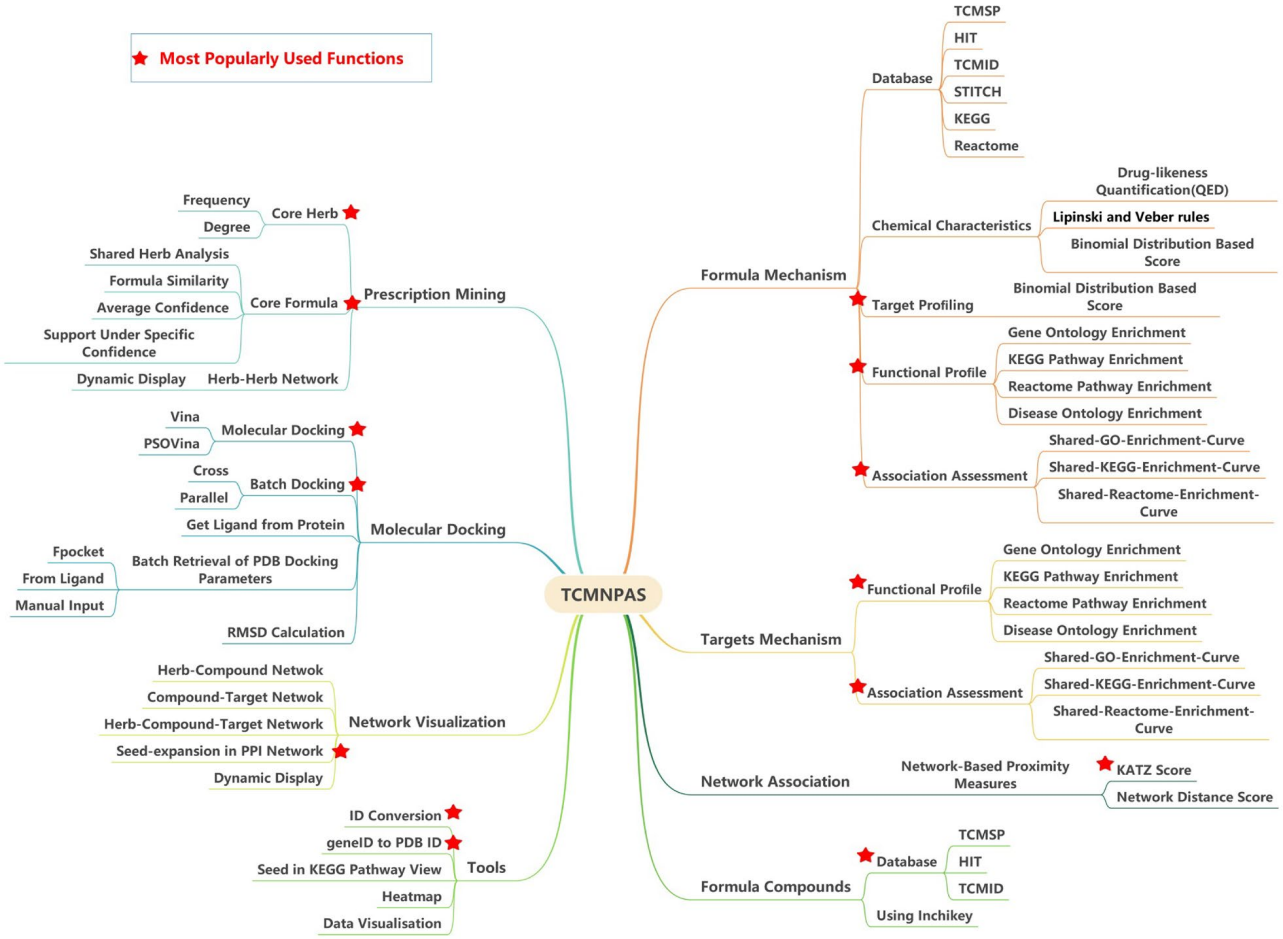
Schematic diagram of the main information architecture

In addition to its extensive data coverage, TCMNPAS provides a wide range of functionalities, making it a valuable resource for researchers. These functionalities include prescription mining, formula mechanism analysis, network-based proximity measures, molecular docking, and network visualization (Fig. 1). Furthermore, TCMNPAS provides several user-friendly tools for data visualization. For a summary of the basic data coverage, please refer to Table 1.

**Table 1:** Data information of TCMNPAS v1.0

Items	Data sources	Count	Total
Herbs	TCMSP [15]	496	1630
	HIT [32]	1062	
	TCMID [16]	1551	
Compounds	TCMSP [15]	13,142	18,090
	HIT [32]	473	
	TCMID [16]	6173	
Herb-Compound Links	TCMSP [15]	15,728	48,554
	HIT [32]	2250	
	TCMID [16]	32,973	
Compound-Target Links	TCMSP [15]	253,057	324,019
	HIT [32]	80,613	
	TCMID [16]	107,416	
	STITCH [33]	240,414	
Herb-Compound-Target Links	TCMSP [15]	3,040,910	3,884,216
	HIT [32]	494,598	
	TCMID [16]	534,107	
	STITCH [33]	3,067,943	
Gene-GO Term Links [37–39]	Biological process	154,265	337,374
	Cellular component	101,134	
	Molecular function	81,975	
Protein–Protein Interactions	HIPPIE [34]	821,849	821,849
Disease-Gene Links	DOSE [40]	222,432	222,432
Gene-Pathway Links	KEGG [35]	34,188	658,649
	Reactome [36]	624,461	

### Analysis process of TCMNPAS

TCMNPAS was purposefully designed to manage extensive TCM data efficiently. It offers 8 main function panels, each serving a distinct purpose in the research and analysis of TCM data.

*Formula Mechanism* This panel investigates the underlying mechanisms of TCM formulas, unraveling the interactions between their various components.

*Targets Mechanism* Here, the platform explores the mechanisms of targets in the context of TCM, shedding light on their roles and functions.

*Network Association* Explore the comprehensive associations between formula targets and disease targets by network-based proximity analysis.

*Formula Compounds* Retrieve the compounds present in specific TCM formulas, elucidating their therapeutic effects and potential synergies.

*Prescription Mining* Utilize network-based techniques to extract valuable information from TCM prescriptions, unveiling hidden patterns and relationships.

*Molecular Docking* Perform molecular docking simulations to assess the interactions between TCM compounds and their target proteins, providing valuable information for drug design.

*Network Visualization* Visualize complex TCM networks to gain insights into their structure and dynamics, aiding in the understanding of the system as a whole.

*Tools* Access a collection of convenient tools for various data visualization and analysis tasks, enhancing the efficiency of researchers' work.

**Fig. 2:**
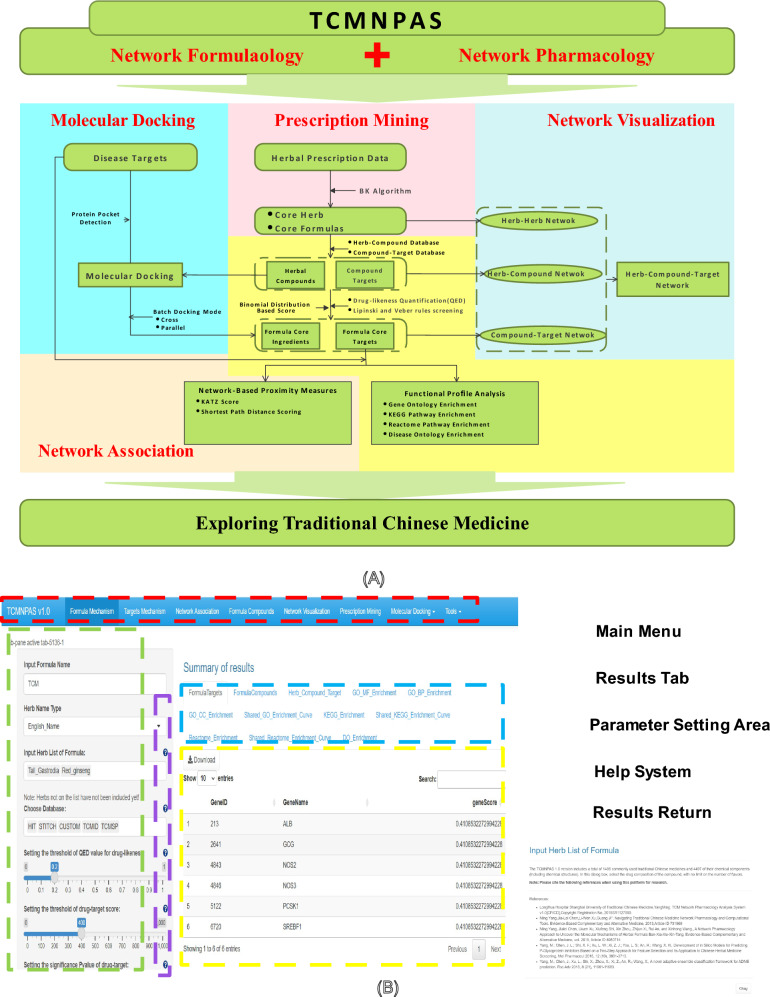
Analysis workflow diagram (**A**) and schematic diagram of the main interface of TCMNPAS (**B**)

The main workflow of TCMNPAS analysis, as depicted in Fig. 2A, shows the logical sequence and connections between the various function panels, enabling a comprehensive approach to TCM research.

### Main interface of TCMNPAS

TCMNPAS presents a user-friendly interface, designed to facilitate efficient and comprehensive TCM data analysis. The interface, as illustrated in Fig. 2B, is thoughtfully divided into five major sections, each serving a specific purpose.

*Main Menu* Located at the top, this section allows users to select the desired panels or analysis items, providing an intuitive starting point for their research.

*Result Tabs* Positioned right side below the main menu, this section enables users to filter and explore the data and results generated from their analysis, ensuring a deeper examination of the findings.

*Parameter Settings Area* Positioned on the left side, users have the freedom to adjust parameters within a desired range, enabling precise and customizable analysis results.

*Help System* To assist new users in their navigation and understanding of the parameter settings, this section offers clear rules and guidelines, streamlining the analysis process.

*Result Return Area* This section provides a clear and concise view of the current analysis data, allowing users to download and save results for future reference and sharing.

Through the cohesive design of these major sections, TCMNPAS streamlines the user experience and enhances the accessibility and convenience of conducting TCM research and analysis.

## Results

### Main functions of TCMNPAS

TCMNPAS is a web-based platform developed using the Shiny framework and HTML. It aims to facilitate comprehensive analysis and research in the field of TCM.

**Fig. 3:**
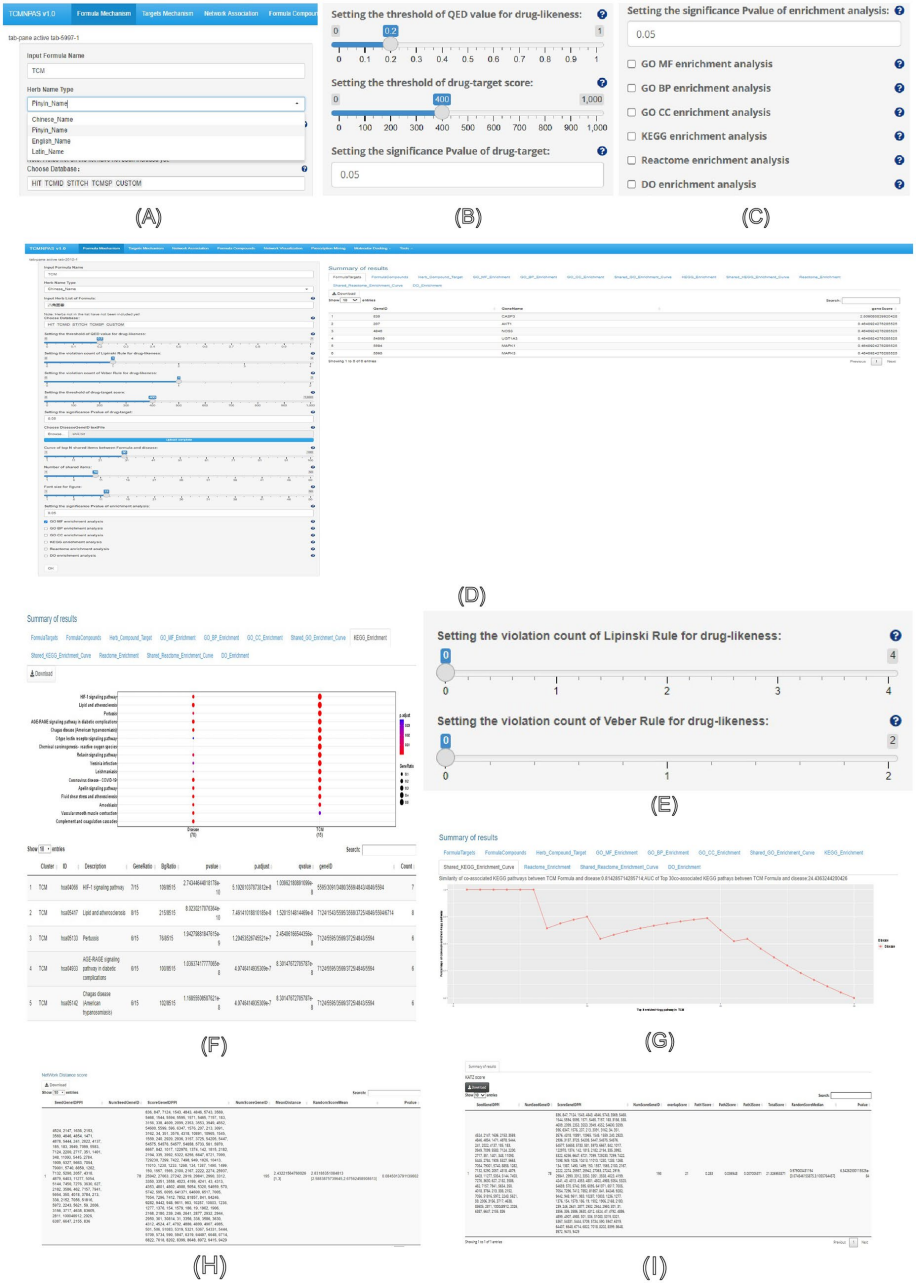
Databases included in TCMNPAS v1.0 for searching herbs, components and targets (**A**), Setting the threshold of QED value for drug-likeness and setting the threshold of drug-target score (**B**), Setting the significance P-value of drug-target (**C**), Herb-Compound-Target (**D**), Setting the violation count of Lipinski Rule and Veber Rule for drug-likeness (**E**), KEGG Enrichment (**F**), Shared-KEGG-Enrichment-Curve (**G**), Network Distance Score (**H**), KATZ Score (**I**)

TCMNPAS offers a range of powerful tools and functions, primarily organized into eight main function panels, as depicted above (Fig. 3).

#### Formula mechanism

The analysis of the chemical characteristics of herbal formulas in TCMNPAS is achieved by inputting a list of herbs, which can be specified using their Chinese name, Pinyin name, English name, or Latin name (Fig. 3A).

In the quest to identify bioactive ingredients within herbal formulas, TCMNPAS employs the quantitative estimate of drug-likeness (QED) presented by Bickerton [41] as a key metric for drug-likeness screening. QED integrates eight essential molecular descriptors for effective analysis of drug-likeness in pharmaceutically active compounds within the formulas [20–22, 41–43], as depicted in Fig. 3B. Additionally, the Lipinski rule [44] and Veber rule [45] are also incorporated for drug-likeness screening. The Lipinski rule assigns values from 0 to 4, representing the number of violations against the rule, with a higher number indicating poorer drug-likeness. According to the “Rule of Five”, a drug-like molecule should have no more than one of the following violations: (1) No more than 5 hydrogen bond donors; (2) No more than 10 hydrogen bond acceptors; (3) Molecular weight no more than 500; (4) LogP no more than 5. Similarly, the Veber rule values range from 0 to 2, indicating violations against the rule, with a higher number signifying reduced drug-likeness. According to the “Rule of Veber”, a drug-like molecule should have no more than one of the following violations: (1) No more than 10 rotatable bonds; (2) Polar surface area of no more than 140 or no more than 12 hydrogen bond donors and acceptors (Fig. 3E).

In TCMNPAS, the identification of core targets for herbal formulas is a crucial step achieved through target profiling of formula ingredients. The determination of core targets relies on a threshold score for a compound-target association, primarily based on the scores obtained from the STITCH database. A higher score in STITCH indicates a stronger association, with a median score of 400. In cases where compound-target pairs lack association scores in other databases, a uniform value of 9999 is assigned [22, 33].

TCMNPAS utilizes a binomial statistical model to facilitate the assessment of target profiling for formulas. This model calculates the probability P(X ≥ k) of a target interacting with k or more active compounds. A target with a smaller P value (e.g., P < 0.05) indicates a significantly larger observed number of interacting compounds, suggesting its role as a core target for the formula [20–22]. The score for a specific target of herbal formula (geneScore) is calcucated by using a numerator that equals the negative logarithm of P(X ≥ k) and a denominator that equals the rank of P(X ≥ k) as follows [20–22]:

1 $$geneScore = \left\{ \begin{gathered} \frac{ - \log (P(X \ge k))}{{Rank(P(X \ge k))}},\,if\;P(X \ge k) < P_{sig} \hfill \\ 0\;,\;\;\;\;\;\;\;\;\;\;\;\;\;\;\;\;\;\;\;\;\;\;\;otherwise \hfill \\ \end{gathered} \right.\;\;\;\;$$

The threshold for identifying core targets of a herbal formula, denoted as Psig, is a user-defined value. This threshold plays a crucial role in the identification of core targets of the herbal formula. Furthermore, the score of a compound, referred to as chemScore, can be determined by averaging its corresponding target scores as follows [20–22]:

2 $$chemScore = \frac{1}{{N_{i} }}\sum\limits_{j = 1}^{{N_{i} }} {geneScore_{j} } \;\;\;\;\;\;\;\;\;\;\;\;\;\;\;\;\;\;\;\;\;\;\;\;\;\;\;\;\;\;\;\;$$

TCMNPAS empowers researchers to characterize the functional profile of formula targets through enrichment analysis. This analysis includes Gene Ontology (GO), KEGG pathways, Reactome pathways, and Disease Ontology (DO). The hypergeometric distribution model is utilized for the enrichment analysis [35, 40]. To ensure statistical significance, the False Discovery Rate (FDR) method is employed to adjust the P values. The enrichment analysis is conducted using the “clusterProfiler package” and “DOSE package” based on R software [20–22, 40, 46] (Fig. 3C).

Additionally, TCMNPAS facilitates the analysis of the association between diseases and formula targets. By providing disease targets, the platform performs a co-association analysis of the enriched terms between formula targets and disease targets. The “Shared-GO-Enrichment-Curve,” “Shared-KEGG-Enrichment-Curve,” and “Shared-Reactome-Enrichment-Curve” options display co-association curves, allowing users to adjust the number of co-associated terms. TCMNPAS provides co-association scores and AUC values for shared term curves, aiding in the evaluation of the degree of association between formula targets and disease targets. This information enables researchers to infer the potential roles of formula targets in disease treatment (Fig. 3D, F-G).

#### Targets mechanism

Researchers utilizing the system have the flexibility to customize the name of the target group and input standard Entrez GeneIDs directly or use text files containing GeneIDs for target group analysis. Furthermore, they have the option to provide text files containing disease-related targets (".txt" or ".csv") with one ID per line, using standard Entrez GeneIDs. When disease targets are inputted, the analysis results will showcase the corresponding molecular mechanisms of the disease targets, allowing for comparison with the inputted target group and highlighting them as “Disease” in the results (Additional file 1: Figure S1).

Upon inputting disease targets, the system presents tabs for “Shared-GO-Enrichment-Curve” and “Shared-KEGG-Enrichment-Curve”, which display co-enrichment curves. Additionally, the “GO-MF-Enrichment,” “GO-BP-Enrichment,” “GO-CC-Enrichment,” “KEGG-Enrichment,” “Reactome-Enrichment,” and “DO-Enrichment” tabs concurrently exhibit co-enriched scatter plots of both the formula and the disease. The co-enrichment terms are displayed in this section for comprehensive analysis.

“Formula Targets”, “Formula Compounds”, “Herb-Compound-Target”, “Shared-GO-Enrichment-Curve”, “Reactome Enrichment”, “Shared-Reactome-Enrichment-Curve” and “DO Enrichment” results are shown in Additional file 1: Figures S2-8.

#### Network association

In the context of TCMNPAS, relevance inference relies on two critical scores: the KATZ score [26] and the network distance score [21, 22]. These scores are applied to assess the relevance between formula targets and disease targets based on their connectivity within the Protein–Protein Interaction (PPI) network integrated by TCMNPAS, derived from the HIPPIE (Human Integrated Protein–Protein Interaction Reference) database [34].

The KATZ score is a relevance score that considers the distance and path between network nodes, with a path score coefficient (Beta) of 0.001. The output of the KATZ score includes various specific score items such as overlap score, Path 1 score, Path 2 score, Path 3 score, Total Score, Random Score Medium, and P-value (Fig. 3H). On the other hand, the network distance score represents the shortest path length in the PPI network. The specific items included in the network distance score output are mean distance, mean random score, and P-value (Fig. 3I).

Both the KATZ score and the network distance score serve as essential metrics for evaluating the relevance between formula targets and disease targets, with higher relevance scores indicating closer proximity between them in the PPI network.

#### Formula compounds

TCMNPAS offers a comprehensive formula compounds retrieval module, encompassing 1630 commonly used herbs and 18,090 compounds (including their chemical structures) [21, 22, 42, 43]. Users can retrieve formula compounds by inputting herb lists with Chinese names, Pinyin names, English names, or Latin names. Additionally, TCMNPAS allows the retrieval of compound information by entering the chemical structure representation of a compound (InChIKey, e.g., ZYGHJZDHTFUPRJ-UHFFFAOYSA-N). This function facilitates the retrieval of information about the compound in various herbs (Additional file 1: Figure S9) [15, 16, 22, 33]. Researchers can further explore the retrieved compounds for their properties or structures by utilizing additional sources such as PubChem [47].

#### Network visualization

In TCMNPAS, researchers can easily visualize the Herb-Compound-Target network. The required network file is available in CSV format, can be downloadable from the “Herb-Compound-Target” tab in the Formula Mechanism module.

The integrated PPI network in TCMNPAS is derived from HIPPIE. By selecting the “Seed-expansion in PPI network” option, corresponding targets are projected onto HIPPIE, leading to the formation of subnetwork outputs [21, 22, 34]. Additionally, the system offers three network types to choose from, with the option to enable PPI network projection and dynamic display (Additional file 1: Figure S10).

#### Prescription mining

**Fig. 4:**
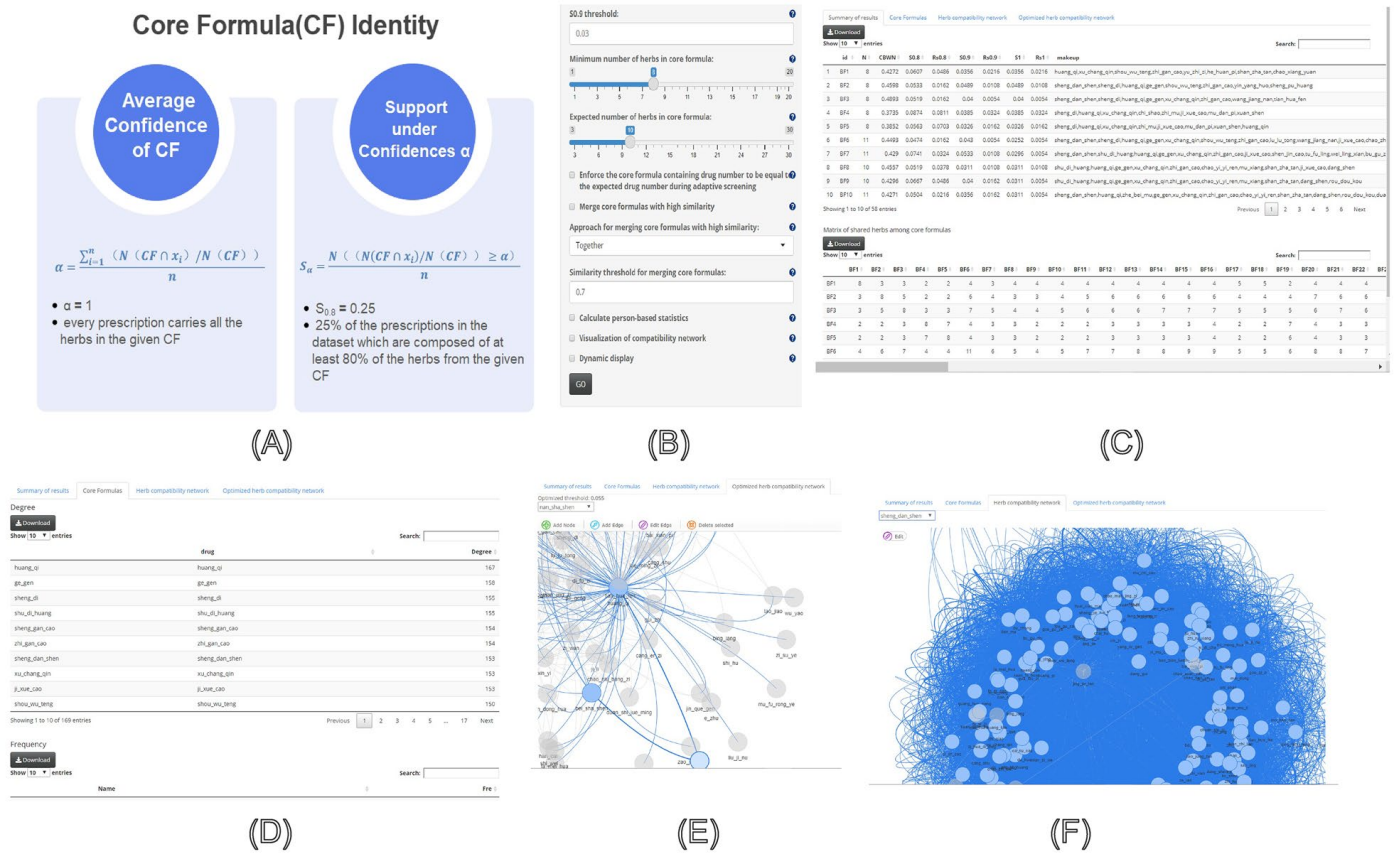
Prescription mining algorithm (**A**), parameter setting area for prescription mining analysis (**B**), Summary of results (**C**), Core formulas (**D**), Herb compatibility network (under optimized threshold) (**E**), Optimized herb compatibility network (**F**)

In recent years, a prescription mining framework based on herb-herb networks has been developed. This framework involves core herbs (combined with network centrality analysis), core herb pairs (combined with the entropy approach), core formulas (combined with the BK algorithm [23–25]), and core effective formulas (combined with the GA algorithm and regression model) (Fig. 4A). Notably, several medication rules/guidelines of renowned TCM experts, such as Professor Liu Jiaxiang (Chinese medical master, an expert in oncology) [48, 49], Professor Tang Hanjun (a mammography expert) [50, 51], Professor Xu Rongjuan (an expert in endocrinology) [52], and Professor Chen Yipin (a nephrology expert) [53, 54], have been effectively summarized.

To expedite the rapid analysis of core herbs, core herb pairs, and core formulas, the platform provides a prescription mining functionality that mainly utilizes the BK algorithm to find the core formulas in herb-herb networks. The basic principle is to find all the maximum cliques based on the recursive procedure for optimizing the candidate-selected herb. The algorithm continuously replaces the herb to continue the search until all herbs have been traversed, thereby obtaining all the maximum cliques in the network. These maximum cliques in the herb-herb network can be considered core formulas.

Individualized treatment in TCM involves formulating therapeutic prescriptions by adding or reducing herbs based on core formulas after syndrome differentiation. Generally, herb-herb networks are weighted undirected networks, where the frequency of herb combinations is used as the edge weight of the network. However, the BK algorithm is only applicable to unweighted networks. Therefore, TCMNPAS performs adaptive binarization on weighted networks before running the BK algorithm and evaluates core formulas based on the two metrics of prescription support and confidence: (1) average confidence of a core formula (α); (2) support under a confidence α, Sα. These two metrics are described in detail elsewhere [55].

In the context of prescription mining analysis using TCMNPAS, researchers need to follow specific steps and set various options for optimal results. The initial step involves uploading prescription data, followed by configuring the S_0.9_ threshold, the minimum number of herbs in the core formula, and the desired number of herbs. This module has certain formatting requirements for the prescription data uploaded by users: (1) The file format should be CSV in UTF-8 encoding; (2) Prescription data should consist of 3 columns [Patient ID (Pid), Visit ID (Vid), and Herb composition]. Vid is used to identify different visit times. If there is no visit ID, please use the same value. Pid is used to identify different patients. Additionally, users can choose from several options, including “Enforce the core formula containing drug number to be equal to the expected drug number during adaptive screening” and “Merge core formulas with high similarity.” The next step is to determine the method for merging highly similar core formulas, set the similarity threshold for merging core formulas, and choosing options such as “Calculate person-based statistics,” “Visualization of compatibility network,” and “Dynamic display” [25, 42, 55, 56](Fig. 4B).

Firstly, users must set the threshold for S_0.9_ support, typically between 0.01 and 0.3. A higher S_0.9_ value indicates higher support for the discovered core formulas; however, it yields fewer amounts. While it's crucial to set the minimum number of herbs in the core formula, one must be careful not to set this number too high, as it could prevent finding core formulas that satisfy the requirements. The binarization threshold also requires optimization during core formula mining. Secondly, users should predefine the desired number of herbs in the core formula, being mindful not to set it too high, as it may result in no core formulas that meet the requirements. Further options include selecting whether the number of herbs in the core formula should be equal to the desired number (maximizing the number of core formulas with the desired herb count) and whether highly similar core formulas should be forcibly merged.

TCMNPAS provides two methods for consolidating highly similar core formulas: “Together,” which merges highly similar core formulas collectively, and “Step,” which incrementally merges highly similar core formulas. The similarity threshold for merging core formulas should be set between 0 and 1, with a recommendation to choose a value greater than 0.6. If the input prescription data includes Vid, selecting the “Calculate person-based statistics” option will facilitate patient-based core formula statistics [25, 55–57].

The input file format must adhere to specific requirements. The prescription data should be in CSV format and include three categories (Pid, Vid, and herb). Pid represents the patient ID, used to identify different patients, while Vid represents the visit ID, used to identify different time points. Herb represents the composition of the prescription. Two considerations are crucial during file preparation: firstly, if there is no visit ID, it should be marked with the same value; secondly, attention should be paid to the standardization of herb names in the prescription composition.

This module provides four essential analysis results, namely “Summary of results”, “Core formulas”, “Herb compatibility network”, and “Optimized herb compatibility network” (Fig. 4C-F).

#### Molecular docking

**Fig. 5:**
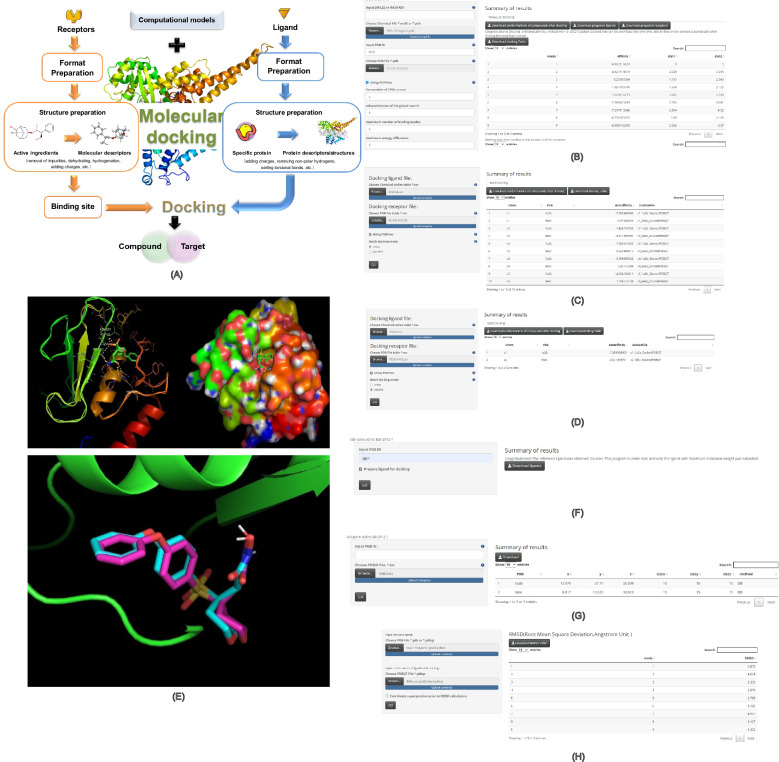
Molecular docking principle diagram (**A**), Parameter setting and result example page for single molecule docking (**B**), Cross batch docking mode (**C**), Parallel batch docking mode (**D**), Molecular docking results (**E**), Extraction of standard ligands from PDB (**F**), Batch retrieval of PDB docking parameters (**G**), RMSD calculation (**H**)

Molecular docking is a critical bioinformatics technique, that plays a significant role in understanding the interaction between molecules (Fig. 5A). This computational method allows researchers to investigate the binding affinity and spatial arrangement of molecules, shedding light on their potential interactions and functional implications. In the TCMNPAS platform, both single-molecule docking and batch docking modes are provided (Fig. 5B). The platform incorporates the Autodock Vina molecular docking module (open-source software, https://vina.scripps.edu/) [27, 28], which supports Vina and PSOVina [29, 30]. The PSOVina algorithm, an optimized version of Autodock Vina, utilizes a hybrid particle swarm optimization algorithm, achieving higher accuracy and speed compared to Autodock Vina [29–31].

To perform molecular docking in TCMNPAS, compounds can be inputted in SMILES or INCHIKEY format. Alternatively, compound files (. mol2 or.pdb) can be uploaded, and the default Vina ligand preparation program [27]will be utilized. The protein structures of specific targets can be obtained and prepared using the “Batch retrieval of PDB docking programs” in the Molecular Docking module [58].

TCMNPAS offers two built-in methods for obtaining protein docking pocket parameters: Fpocket [59] and ligand-based. When using the ligand-based method, it's necessary to input the protein with its native ligand to extract corresponding pocket parameters from the ligand’s position. The platform also facilitates extraction of native ligands from PDB files. For Fpocket prediction, version 2.0 of the Fpocket program is utilized. Alternatively, users can opt to manually input parameters such as center_x, center_y, center_z, size_x, size_y, and size_z. The platform additionally supports batch retrieval of PDB docking parameters and root mean square displacement (RMSD) calculations (Fig. 5F-H).

In the batch docking mode, users can choose between two options: (1) Cross mode [each ligand docked with each receptor (Fig. 5C)], and (2) Parallel mode [ligands paired with receptors for docking (Fig. 5D)]. In parallel mode, it is essential to ensure that the number of ligands matches the number of receptors. TCMNPAS supports multiple scoring functions to evaluate the affinity and stability of molecular docking, taking into account various factors such as binding energy and steric hindrance to improve the reliability of docking results. After docking is completed, TCMNPAS generates a detailed docking report, including binding modes and scoring values for each molecule with the target protein. Users can visualize and analyze the docking results using the provided tools to gain insights into the binding modes and activity of the molecules. Additionally, the docking protocol can be validated in TCMNPAS by calculating the RMSD of redocked poses of native ligands. The final molecular docking result is presented in Fig. 5E.

As an example of ligand docking in TCMNPAS, quercetin was selected [60]. Quercetin’s “Standard SMILES” was retrieved from the PubChem database, and the “Protein Data Bank (PDB) ID” for Akt (3O96) was obtained from the RCSB PDB database (https://www.rcsb.org/). Subsequently, both pieces of information were input into the molecular docking module of TCMNPAS to analyze the binding affinity of quercetin towards Akt1. The results demonstrated a strong binding affinity of − 8.9 kJ/mol, indicating a favorable interaction between quercetin and Akt1 [61].

#### Tools

The TCMNPAS system offers a range of valuable tools to facilitate various analyses and data visualizations.

*ID Conversion* The “ID Conversion” tool offers batch conversion capabilities, allowing users to convert specified variables between Entrez Gene IDs and Gene Symbols, both from entrez gene ID to gene SYMBOL and vice versa. (Additional file 1: Figure S11).

*Gene ID to PDB ID* With the “gene ID to PDB ID” tool (Additional file 1: Figure S12), users can input gene IDs, and the system will provide corresponding PDB IDs for further exploration.

*Seed in KEGG Pathway* The “Seed in KEGG pathway” tool [62] (Additional file 1: Figure S13) allows users to input their desired pathway ID, and the system will display the corresponding KEGG pathway diagram, assisting in pathway analysis.

*Heatmap* The “Heatmap” tool [63] (Additional file 1: Figure S14) provides customizable options, and offers flexible customization options for personalized heatmap visualization.

*Data Visualization* The “Data Visualization” tool (Additional file 1: Figure S15), allows users to select plot type, and adjust the size of icons, text font size, and font style for visualizing various datasets based on specific user preferences.

## Applications of TCMNPAS

**Fig. 6:**
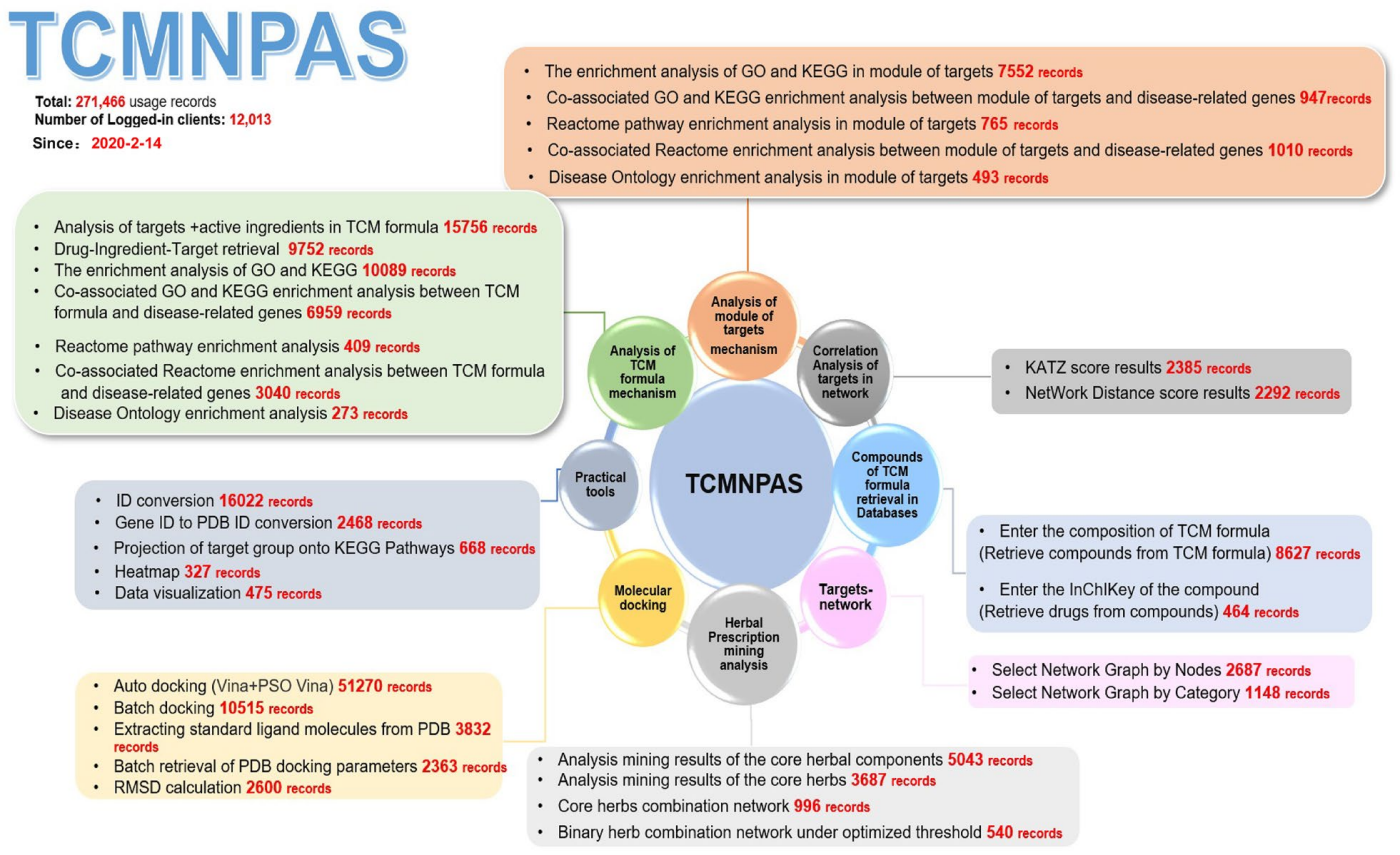
Overview of the main functions of TCMNPAS v1.0 (including usage frequency and number of users)

The TCMNPAS system possesses a wide application range, encompassing NF and NP analysis of TCM. The system has proven to be highly beneficial, with over 270,000 usage records and 12,000 researchers benefiting from TCMNPAS, according to backend data statistics (Fig. 6). Researchers have extensively utilized TCMNPAS for in-depth analysis and validation of the therapeutic mechanisms of TCM in the treatment of specific diseases. This demonstrates the significant impact and value of the TCMNPAS system in advancing research in the field of TCM.

### Network construction and analysis of targets from TCM formula association with disease’s targets

TCMNPAS, as highlighted by Shiyu Ma et al. [64], is a powerful analytical platform for NP analysis on the mechanism of DCXF (Da Chuan Xiong Fang) intervention in migraine. The study demonstrated that there was a significant similarity of 0.79 between the targets of DCXF and the disease genes associated with migraines. Additionally, the co-enrichment curve showed an AUC value of 17.1, indicating a strong correlation. Furthermore, the co-enrichment analysis of GO pathways revealed a robust association between the targets of DCXF and migraine-related genes. Metabolomics results also indicated that DCXF had an intervention effect on the alterations of endogenous neurotransmitters in both serum and brain tissues caused by migraines, helping to restore them to a normal state similar to that before the onset of migraines. These findings provide valuable insights into the potential therapeutic mechanisms of DCXF in treating migraines.

One study was finished by Jinbiao He et al. [65] applied TCMNPAS to analyze the correlation between the effect of Kuijie Kang (KJK) and ulcerative colitis (UC). It was found that there was a significant overlap between the targets of KJK's active ingredients and the targets associated with UC (P = 0.000191). The co-enrichment GO similarity between the targets of KJK and UC was 0.74. In the top 30 pathways, the AUC value in the co-enrichment curve was 6.61, providing further evidence for the application value of KJK in UC.

In another study finished by Jinbiao He et al. [66], the effect of *Poria cocos* (Schw.) Wolf. (Fu Ling) extract on metabolic dysfunction-associated fatty liver disease (MAFLD) through the FXR/PPARα-SREBPs pathway was investigated. The co-correlation between MAFLD targets and Fu Ling targets was studied by TCMNPAS. The results showed that the similarity of co-enriched GO terms between targets from Fu Ling active ingredients and MAFLD targets was 0.87, with an AUC of 4.84 for the top 30 co-enriched GO terms. The similarity of co-enriched pathways between Fu Ling and MAFLD was 0.78, with an AUC of 4.47 for the top 30 pathways.

In the study conducted by Junmin Wang et al. [67], TCMNPAS was utilized as a valuable tool for NP analysis to investigate the specific mechanism of Qinggan Huoxue Fang (QGHXF) in alleviating alcoholic liver disease (ALD). The results suggested that QGHXF may improve ALD by inhibiting the PI3K/AKT signaling pathway.

In a study finished by Lingjian Guo et al. [68], TCMNPAS was used to elucidate the mechanism of Siteng Fang (STF) in reversing multi-drug resistance in gastric cancer (GC) cells. The study utilized TCMNPAS to identify the effective components and targets of STF and genes related to GC. Subsequently, molecular docking using the PSOVina mode was performed, indicating a favorable binding efficacy between the effective components and targets.

### Extensively applied in molecular docking/batching docking

Given the demanding requirements and time-consuming nature of molecular docking experiments, TCMNPAS offers a more convenient online platform for conducting docking analysis.

In a comprehensive study by Suxian Liu et al. [69], the therapeutic effects of *Curcuma longa* L. (Jiang Huang) on ulcerative colitis (UC) were systematically elucidated. Molecular docking between the core components of Jiang Huang and core targets in UC was finished by TCMNPAS. From the docking results, 24 proteins were selected for further network analysis. Ultimately, 12 active ingredients containing 148 target genes were identified from Jiang Huang, and potential targets for treating UC were selected from the overlapping targets between UC and Jiang Huang, totaling 54 targets.

The potential mechanism of *Alpinia oxyphylla* Miq. (Yi Zhi) in combating Alzheimer's disease (AD) was explored by Rong-Rong Zhen et al. [70]. TCMNPAS was utilized to investigate the targets associated with Yi Zhi and AD. Molecular docking was performed by TCMNPAS, and the results revealed that most of the active ingredients could interact with three target proteins, PPARG, ESR1, and AKT1. Specifically, Salicin and Icariside II exhibited a stronger binding rate towards PPARG, ESR1, and AKT1. Salicin and Icariside II formed hydrogen bonds and carbonyl interactions with Lys14 and Arg86 residues of AKT1.

### Combination with network formulaology and NP

In the study conducted by Shiyu Ma et al. [71], TCMNPAS was used to explore the TCM formulas intervention by rectal cancer patients with the syndrome of “Qi and Blood” deficiency. Distinct core prescriptions for the advanced stage, chemotherapy stage, and recovery stage were identified and elucidated, aiming to investigate their potential mechanisms in treating rectal cancer. The BK algorithm was used to extract three core prescriptions, and the shared herbs in these core prescriptions were identified, including *Curcuma phaeocaulis* Val. (E Zhu), *Astragalus membranaceus* (Fisch.) Bge. (Huang Qi), *Scleromitrion diffusum* (Willd.) R. J. Wang (Bai Hua She She Cao), and Fu Ling. These combinations accounted for 36.4% of Core Prescription I, 36.4% of Core Prescription II, and 50% of Core Prescription III. E Zhu and *Coix lachryma-jobi* L.var.*mayuen*(Roman.) Stapf (Yi Yi Ren) appeared most frequently in the core prescriptions. Additionally, active ingredients, targets, activated signaling pathways, and biological functions of core prescriptions were explored, and the binding energy analysis with target proteins was performed using the batch docking function in TCMNPAS, indicating the crucial roles of these active ingredients in the treatment of rectal cancer. Furthermore, these studies may help find hub genes that affect the tumor microenvironment and survival. The combination of network formulaology and NP may help elucidate the relationship between herbs acting on “Zheng” (syndrome) and diseases, thus expanding the understanding of TCM mechanisms.

## Discussion

### Advantages and innovations

TCMNPAS platform offers all its functionalities for open-source by users and researchers, with over 270,000 usage records. It focuses on Network Formulaology, NP analysis, etc. The platform is available in both Chinese and English versions, making it more convenient for scholars from both domestic and international communities.

In previous studies on Network Formulaology, many researchers have focused on the data mining and analysis of core prescriptions and core herbs. They have explored various core prescriptions and core herbs intervened on diseases such as Wen Disease and Shanghan Lun mentioned in the classic TCM literature [72], core prescriptions applied during the COVID-19 pandemic [73], core prescriptions treated on rectal cancer [74] and chronic liver disease [75]. The research analysis provided by the TCMNPAS platform emphasizes the integration of NF and NP.

TCMNPAS highlights the analysis mode of core prescription with core-herb-target by combining multiple algorithms to explore the association and compatibility patterns between effective ingredients of TCMs formulas and their targets in prescriptions. By analyzing the co-association between disease genes and targets of effective ingredients, provides insights into the associations and synergies between effective ingredient targets and diseases. This approach can predict and elucidate the potential mechanisms and advantages of prescriptions in the treatment of specific diseases to some extent. The platform is also capable of conducting enrichment analysis, elucidating the biological process, molecular functions, cell components, signal pathways, and metabolic pathways through which effective ingredients may affect diseases.

In addition, the platform integrates high-performance and cost-effective molecular docking capabilities, supporting online batch docking in two modes, which saves analysis time and improves research efficiency for users and researchers. When using the molecular docking module, the platform can directly obtain the required target molecules, complete the corresponding pre-processing steps, and obtain docking pocket information (with the option to set the pocket acquisition mode). This enables researchers to optimize the docking process conveniently and flexibly.

Furthermore, we closely attend to researchers' feedback and continuously maintain system upgrades, as well as address any problems/bugs that may arise during the development and application of the platform. We have not only synchronized and updated with the HIPPIE and KEGG but also have introduced important features such as significance scoring of key target genes and compounds based on the binomial distribution.

### Limitations and challenges

In the process of research on TCM, numerous NP analysis platforms have emerged, each with its unique advantages and characteristics. And their applications and developments have contributed to the role of NP in TCM research. Currently, there are certain limitations in the process of NP analysis of TCM. One limitation lies in the data quality of public databases, which can be influenced by various factors. These factors include differences in experimental instruments, rapid updates in techniques and design methods, as well as the absence of standardized data formats and experimental designs. On the other hand, the exact spectrum of compounds of TCM herbs is not defined, resulting in bias and incomplete inferences. Furthermore, the drug target database has shortcomings, as drug targets are not limited to proteins but also encompass RNA and DNA, among others. Quantifying the therapeutic effects of TCM targets on diseases remains a significant challenge due to this complexity. In addition, the use of different extraction techniques, such as alcohol extraction and water extraction, combined with high-throughput omics technology analysis, yields a variety of extracted chemicals from the herbs. It is essential to consider the bioavailability of these compounds and the influence of omics technology in future research endeavors. Therefore, it is essential to propose solutions or methods directly after addressing the challenges and limitations. Specifically, the updated and validated data sources, along with quantitative network pharmacology techniques and methodologies, are necessary to overcome these constraints effectively. By tackling these challenges, researchers can improve the accuracy and reliability of NP analysis in TCM, leading to a more comprehensive understanding of TCM’s therapeutic effects and facilitating its integration into modern healthcare practices.

For TCMNPAS, we will further expand the data sources, primarily including the chemical constituents of TCM, data on biological activity evaluations, and information on drug targets. In addition, it is also worth considering the introduction of other relevant databases, such as drug metabolism, drug toxicity, adverse effects, etc. This will provide a more comprehensive understanding of the pharmacological characteristics of TCM formulas. Meanwhile, optimization of the model algorithms will be conducted to enhance both prediction accuracy and efficiency. TCMNPAS will integrate clinical practice and trial data to validate and refine the pharmacological characteristics of TCM formulas.

Lastly, TCMNPAS may strengthen data sharing and platform connection by integrating and sharing data with other relevant platforms and databases, accelerating the development of NP research on TCM and facilitating the modernization of TCM.

## Conclusion and perspective

With the rapid development of NP in the past decade, the NP analysis platforms will provide greater convenience and more reliable predictive guidance for the development of TCM. The development of TCMNPAS plays a crucial role in promoting data sharing and resource integration. It offers a more comprehensive and reliable data resource for research in NP, thereby enhancing the quality of studies. As a result, TCMNPAS provides more adequate and scientifically supported data, which proves invaluable for drug development and clinical practice.

In the future, researchers should pay greater attention to omics research in TCM when establishing NP analysis platforms. By integrating clinical experimental studies, it is essential to develop more comprehensive and accurate databases of TCM and sophisticated tools for molecular mechanism analysis. These efforts will drive the modernization and development of TCM.

### Supplementary Information

**Additional file 1: Table S1.** Overview of TCMNPAS and other TCM analysis platforms. **Figure S1.** Target Mechanism. **Figure S2.** Formula Mechanism-Formula Targets. **Figure S3.** Formula Mechanism-Formula Compounds. **Figure S4.** Formula Mechanism-GO-MF-Enrichment. **Figure S5.** Formula Mechanism-Shared-GO-Enrichment-Curve. **Figure S6.** Formula Mechanism-Reactome Enrichment. **Figure S7.** Formula Mechanism-Shared-Reactome-Enrichment-Curve. **Figure S8.** Formula Mechanism-DO Enrichment. **Figure S9.** Formula Compounds. **Figure S10.** Network Visualization. **Figure S11.** Tools-ID Conversion. **Figure S12.** Tools-Seed in KEGG pathway. **Figure S13.** Tools-Heatmap. **Figure S14.** Tools-Data Visualization. **Figure S15.** Tools-Data Visualization results.

## Data Availability

The TCMNPAS package is freely available under the GPL-3.0 license from GitHub (https://github.com/yangpluszhu/tcmnpas), and the Shiny app is freely available at http://54.223.75.62:3838/.

## Abbreviations

TCM: Traditional Chinese Medicine
NF: Network formulaology
NP: Network pharmacology
TCMNPAS: Traditional Chinese Medicine Network Formulaology and Pharmacology Analysis System
QED: Quantitative estimate of drug-likeness
GO: Gene Ontology
DO: Disease Ontology
FDR: False Discovery Rate
PPI: Protein–Protein Interaction
HIPPIE: Human Integrated Protein–Protein Interaction Reference
BK: Bron-Kerbosch algorithm
Vid: Visit IDs
Pid: Patient IDs
RMSD: Root mean square displacement
PDB: Protein Data Bank
DCXF: Da Chuan Xiong Fang
KJK: Kuijie Kang
UC: Ulcerative colitis
MAFLD: Metabolic dysfunction-associated fatty liver disease
QGHXF: Qinggan Huoxue Fang
ALD: Alcoholic liver disease
STF: Siteng Fang
GC: Gastric cancer
UC: Ulcerative colitis
AD: Alzheimer's disease
