# Supplementary material for: TCMNPAS: a comprehensive analysis platform integrating network formulaology and network pharmacology for exploring traditional Chinese medicine
Source: Chin Med. 2024 Mar 22;19:50. doi: 10.1186/s13020-024-00924-y (PMC10958928; doi:10.1186/s13020-024-00924-y)
Supplement: Supplementary file 1 — Additional file 1: Table S1. Overview of TCMNPAS and other TCM analysis platforms. Figure S1. Target Mechanism. Figure S2. Formula Mechanism-Formula Targets. Figure S3. Formula Mechanism-Formula Compounds. Figure S4. Formula Mechanism-GO-MF-Enrichment. Figure S5. Formula Mechanism-Shared-GO-Enrichment-Curve. Figure S6. Formula Mechanism-Reactome Enrichment. Figure S7. Formula Mechanism-Shared-Reactome-Enrichment-Curve. Figure S8. Formula Mechanism-DO Enrichment. Figure S9. Formula Compounds. Figure S10. Network Visualization. Figure S11. Tools-ID Conversion. Figure S12. Tools-Seed in KEGG pathway. Figure S13. Tools-Heatmap. Figure S14. Tools-Data Visualization. Figure S15. Tools-Data Visualization results. [file 13020_2024_924_MOESM1_ESM.docx]

**Supporting Information**

**TCMNPAS: A Comprehensive analysis platform integrating network formulaology and network pharmacology for exploring traditional Chinese medicine**

Yishu Liu^a^, Xue Li^a^, Chao Chen ^a^, Nan Ding ^a^, Peiyong Zheng^a^, Xiaoyun Chen^a^, Shiyu Ma^b^*, Ming Yang^a^*

^a^ LongHua Hospital Shanghai University of Traditional Chinese Medicine, Shanghai 200032， China

^b^ Ruijin Hospital Affiliated to Shanghai Jiaotong University School of Medicine, Shanghai 200025, China

**Contents**

**Table S1** Overview of TCMNPAS and other TCM analysis platforms

**Figure S1** Target Mechanism

**Figure S2** Formula Mechanism-Formula Targets

**Figure S3** Formula Mechanism-Formula Compounds

**Figure S4** Formula Mechanism-GO-MF-Enrichment

**Figure S5** Formula Mechanism-Shared-GO-Enrichment-Curve

**Figure S6** Formula Mechanism-Reactome Enrichment

**Figure S7** Formula Mechanism-Shared-Reactome-Enrichment-Curve

**Figure S8** Formula Mechanism-DO Enrichment

**Figure S9** Formula Compounds

**Figure S10** Network Visualization

**Figure S11** Tools-ID Conversion

**Figure S12** Tools-Seed in KEGG pathway

**Figure S13** Tools-Heatmap

**Figure S14** Tools-Data Visualization

**Figure S15** Tools-Data Visualization results

**Table S1 Overview of TCMNPAS and other TCM analysis platforms**

| **Platform** | **Description** | **Data Resources** | **Main Functions** | **Type** | **Website** |
| --- | --- | --- | --- | --- | --- |
| TCMNPAS v1.0 | A systematic analysis platform integrating network formulaology and network pharmacology, incorporating multiple resources and a range of functions designed for automated analysis implementation. | TCMSP  TCMID  HIT  STITCH  GO  KEGG  Reactome  DO  HIPPIE | - Formula Mechanism - Targets Mechanism - Network Association - Formula Compounds - Network Visualization - Prescription Mining - Molecular Docking - Tools | Free | Chinese/English：<http://54.223.75.62:3838/>  R package:  https://github.com/yangpluszhu/tcmnpas |
| ETCM v2.0(1) | The Encyclopedia of Traditional Chinese Medicine | The TCM formula and Chinese patent drug information  The Chinese medicinal materials information  The ingredient information  The target information  The disease information | - Retrieval of syndromes, TCM formulas, Chinese patent drugs, Chinese medicinal materials and ingredients, and herbal targets - Target identiﬁcation based on drug similarity evaluation - Molecular mechanisms of TCMs - Clinical repositioning analysis | Free | http://www.tcmip.cn/ETCM2/front/#/ |
| HIT v2.0(2) | An enhanced platform for Herbal Ingredients’ Targets | Herbs  Herbal Ingredients  Biological Targets  Micro RNA Targets | - Retrieval of herbs, ingredients, and targets | Free | http://hit2.badd-cao.net/ |
| TCMIP v2.0(3) | Integrative Pharmacology-based Research Platform of Traditional Chinese MedicineMainly includes five major database resources from the ETCM（Encyclopedia of Traditional Chinese Medicine） | Chinese herbal medicine database  Chinese herbal formulation database  Chinese herbal component database  Chinese herbal medicine target database  Disease-related molecular database | - Disease-related molecular set and its functional exploration - Identification and functional analysis of syndrome-related molecules - Identification and functional analysis of TCM pharmacological-related molecules - Prediction and functional analysis of targets in TCM (including formulations) - Analysis of prescription medication rules - Exploration of the network of TCM - Reverse search for TCM (including formulations) | Payware | <http://www.tcmip.cn/TCMIP/index.php/Home/Login/login.html> |
| BATMAN-TCM(4) | Predict/query the potential targets of TCM ingredients and then perform functional analysis on these targets, including GO terms, KEGG pathways, and OMIM/TTD disease enrichment analysis. | DrugBank  KEGG  TTD  TCMID | - Prediction of potential targets for TCM components - Functional analysis of targets, including biological pathways, gene ontology terms, and disease enrichment analysis - Visualization of the network of components-targets-pathways/diseases and KEGG biological pathways - Comparative analysis of multiple TCMs | Free | <http://bionet.ncpsb.org/batman-tcm>/ |
| SymMap(5) | SymMap combinesTCM with modern medicine through intrinsic molecular mechanisms and external symptom maps, providing abundant information on Chinese medicinal materials/ingredients, targets, and clinical symptoms and diseases for herb screening purposes. | The Chinese Pharmacopoeia (2015)  TCMSP  TCMID  TCM-ID  UMLS  HIT  HPO  DrugBank  NCBI  OMIM  MeSH  Orphanet | - TCM information search - TCM - TCM syndrome - Western medical symptoms - Components - Targets - Disease association network - Querying the components and corresponding targets of TCM - Querying information about TCM syndrome, Western medical symptoms, components, targets, diseases, etc. | Free | <http://www.symmap.org/> |
| HERB(6) | It is a natural herb database platform that integrates high-throughput experimental data and reference mining data. The database provides browsing, retrieval, viewing, and downloading functions for TCMs, active ingredients, target genes, diseases, high-throughput experiments, and reference mining data. | GEO high-throughput experiments  PubMed  TTD  DisGeNet  HPO | - Retrieval of TCM and active ingredient information - **Obtaining high-throughput experimental data of TCM and its components** - **Differential expression analysis and functional enrichment analysis** - **Data mapping of TCM/components with modern drugs** - Quick search for literature related to TCM/components | Free | <http://herb.ac.cn/> |
| ITCM(7) | **The largest herb transcriptome platform based on active ingredients of TCMs. The first high-quality transcriptomics database and online platform related to TCMs, including 496 active ingredients from unified high-throughput RNA-Seq data.** | CAS  PubChem  Molecular Signatures Database (v7.5.1)  KEGG  BIOCARTA  REACTOME  WikiPathways  CMap  SYMMAP  TCMSP  ETCM  NPASS | - Gene expression visualization - Query of information related to TCM - Rapid screening of active ingredients of TCM for specific diseases - Obtaining high-quality transcriptional transcriptome data of active ingredients of TCM | Free | <http://itcm.biotcm.net>. |
| TCMSID(8) | A Traditional Chinese Medicine Simplified Integrated Database, which contains 499 herbs registered in the Chinese pharmacopoeia with 20015 ingredients including comprehensive annotations, was developed to achieve TCM Simplification. In this database, several key ingredients can be screened as representatives of the whole TCM to capture potential targets via implementing multi-tool target prediction. Therefore, Networks among TCM herbs, formulations, ingredients, potential targets can be constructed to facilitate clarifying function and mechanisms of the TCM. | TCM@Taiwan  HIT  TCMSP  TCMID2.0  ETCM  NPASS  NPACT | - Constructed a Traditional Chinese Medicine Simplified Integrated Database containing 20015 herbal ingredients; - Evaluated key herbal ingredients that exert pharmacological effects; - Evaluated the main active ingredients in traditional Chinese medicine; - Evaluated 14 kinds of ADME/T properties for all ingredients; - Evaluated the structural reliability of all ingredients - Provided specific structure category of each ingredient; - Evaluated 8 commonly used physicochemical properties; - Provided potential targets of ingredients by multi target prediction tools; - Constructed the networks between herbs and potential targets | Free | <https://tcm.scbdd.com/> |
| SuperTCM(9) | SuperTCM is a comprehensive database that covers the aspects of Traditional Chinese Medicine (TCM) derived from medicinal plants. It encompasses synergies in pharmaceutical recipes up to molecular ingredients. By tracing all Chinese plant-based drugs back to their source, i.e., their plant of origin, it mapped the drugs carefully, distinguishing common non-scientific plant names versus scientific names. Search for non-plant drugs, for common versus Chinese pharmaceutical names, as well as for disease names are additional features. It provides a compound similarity search through a connection to well-established compound database. Last but not least, it also projected the information on combinations of Chinese drugs in traditional recipes onto KEGG pathways. | SymMap  CMAUP  ETCM  TCMID 2.0  HIT  YaTCM  TCMSP  TCM-Mesh  TCM Database@Taiwan  BATMAN-TCM  TCMAnalyzer  TCM-ID  TCMGeneDIT  TM-MC | - Ingredient similarity search by connecting with the pubchem database. - Reliable ingredient-target interactions - Information on diseases (ICD-10-CM) - Biological pathways - KEGG Global Maps mark the ingredients and targets of a simple drug (a processed medicinal or “herb”) and mixed “herbs” (or recipes), which present on the maps as an overview of how they affect the pathways | Free | <https://tcm.charite.de/supertcm/> |


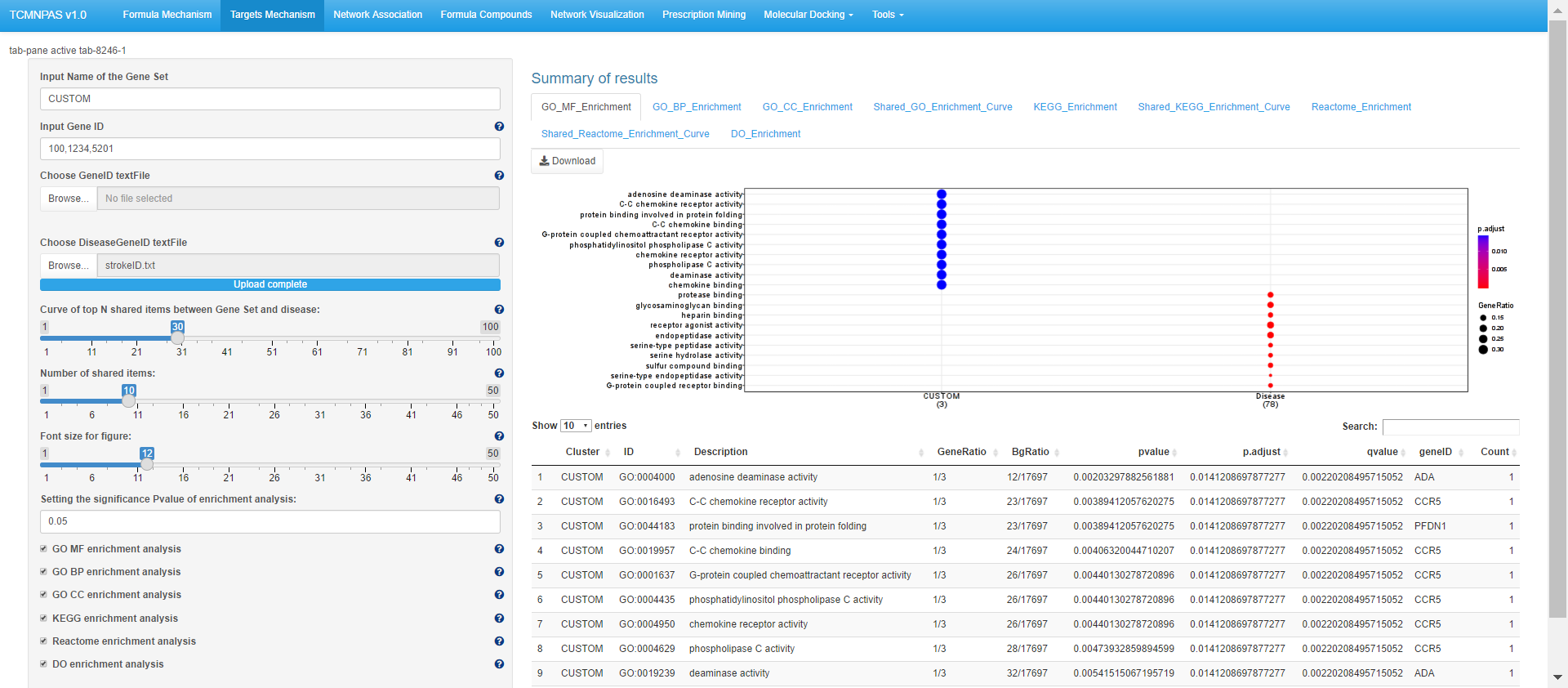


Figure S1Target Mechanism


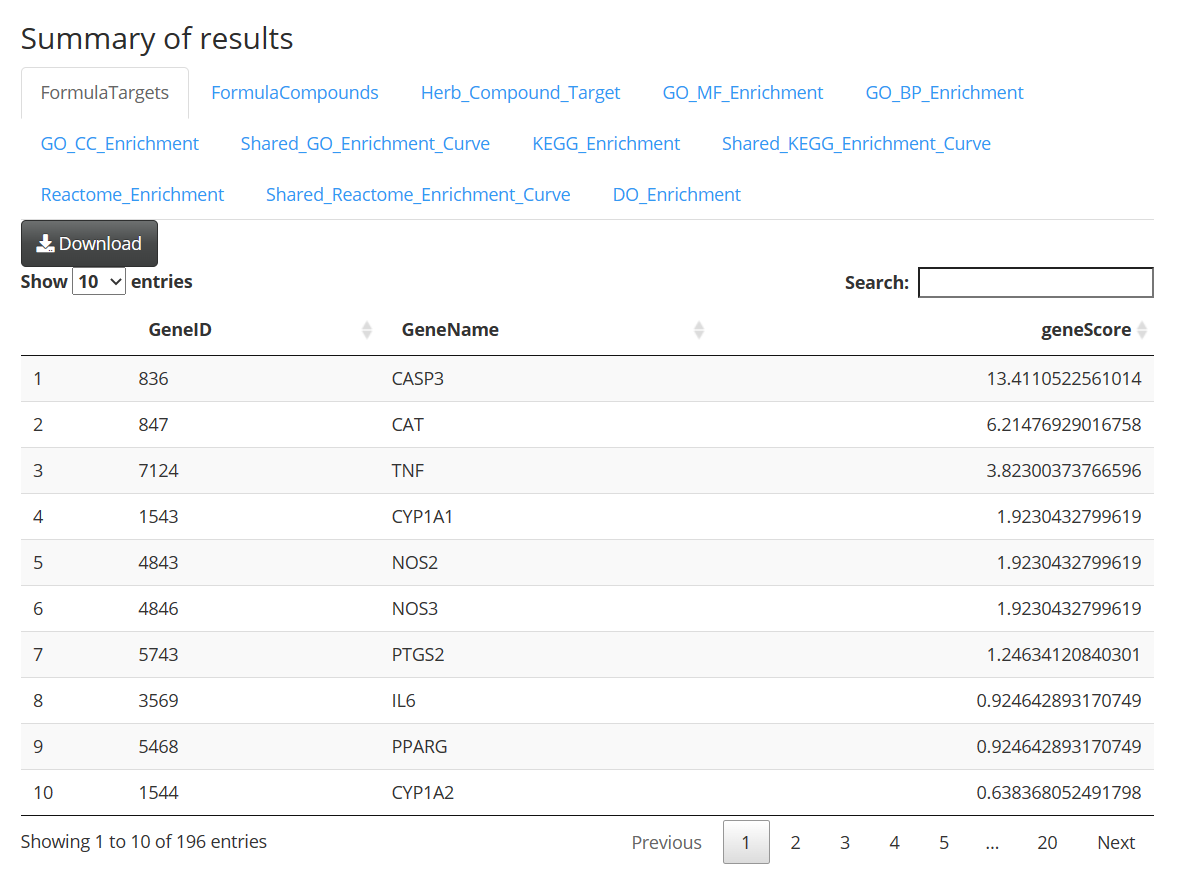


Figure S2 Formula Mechanism-Formula Targets


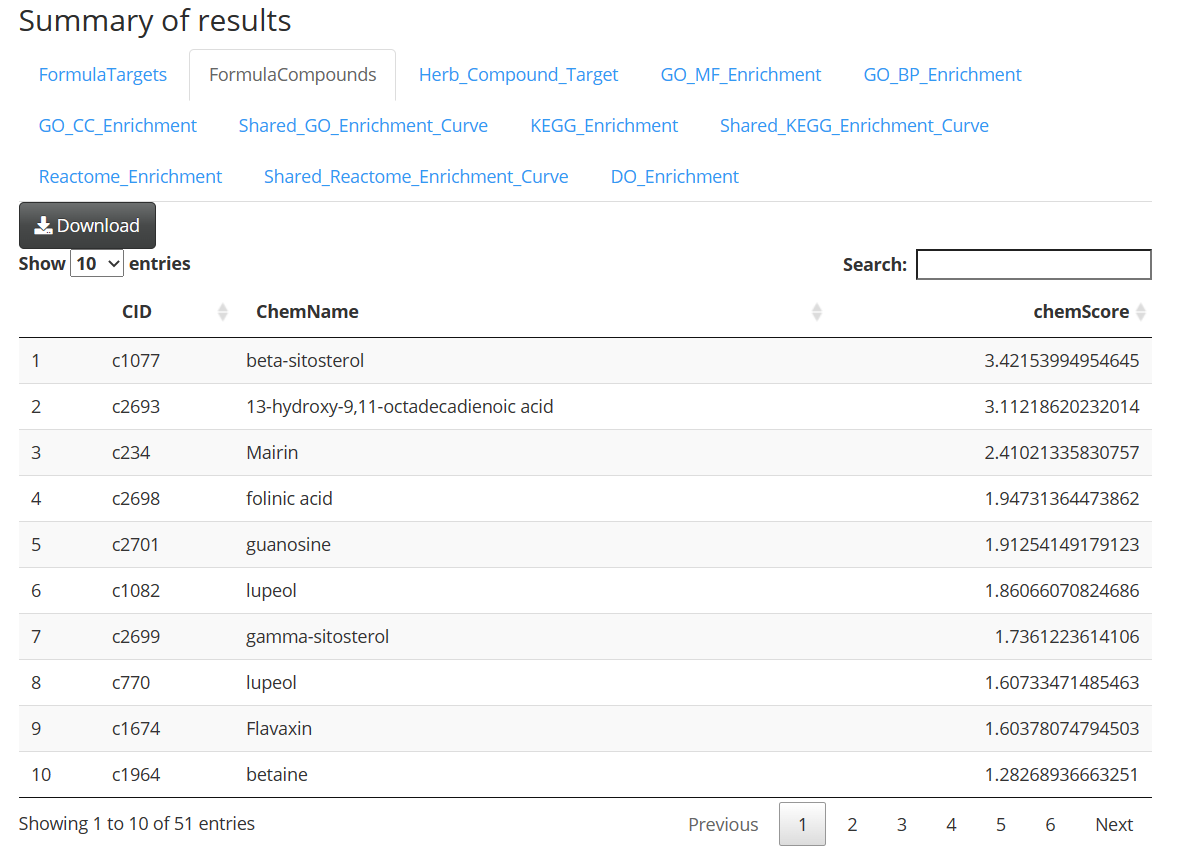


Figure S3 Formula Mechanism-Formula Compounds


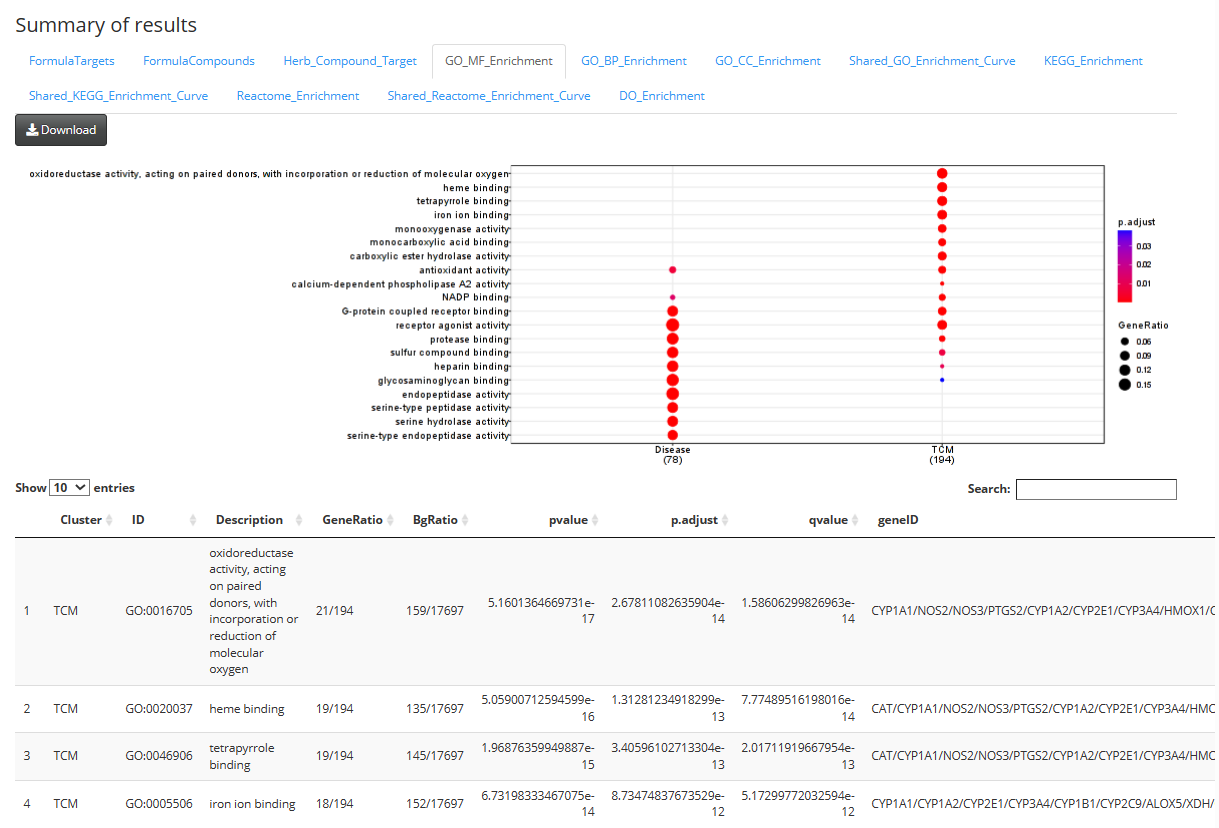


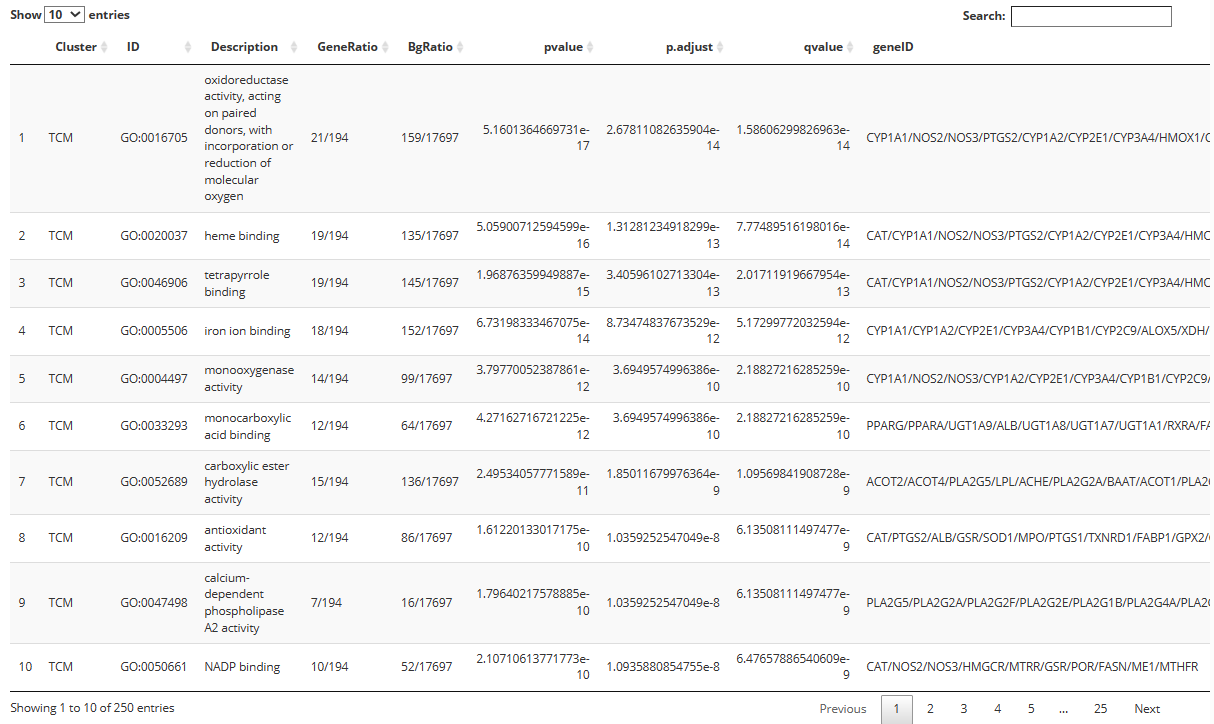


Figure S4 Formula Mechanism-GO-MF-Enrichment


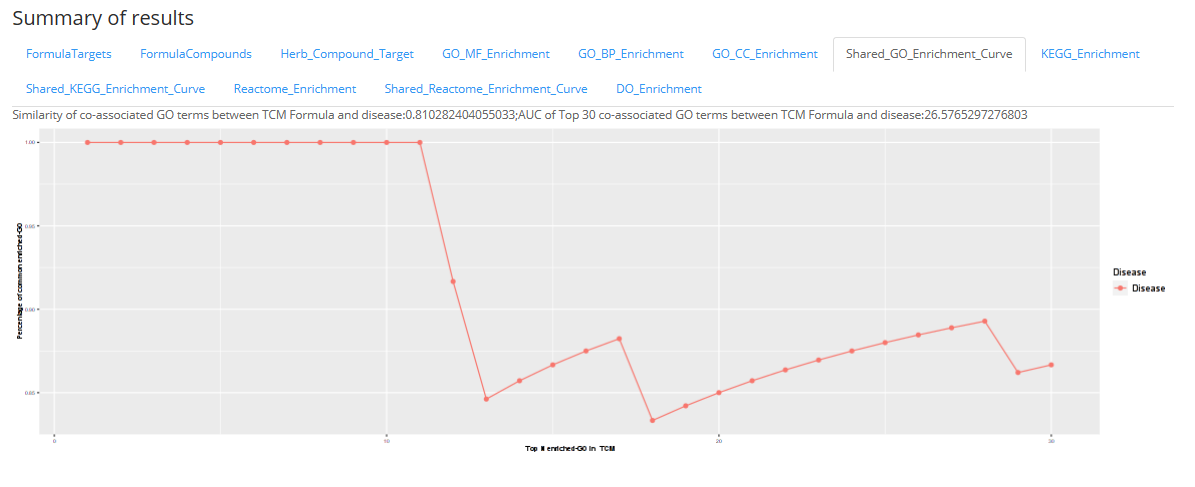


Figure S5 Formula Mechanism-Shared-GO-Enrichment-Curve


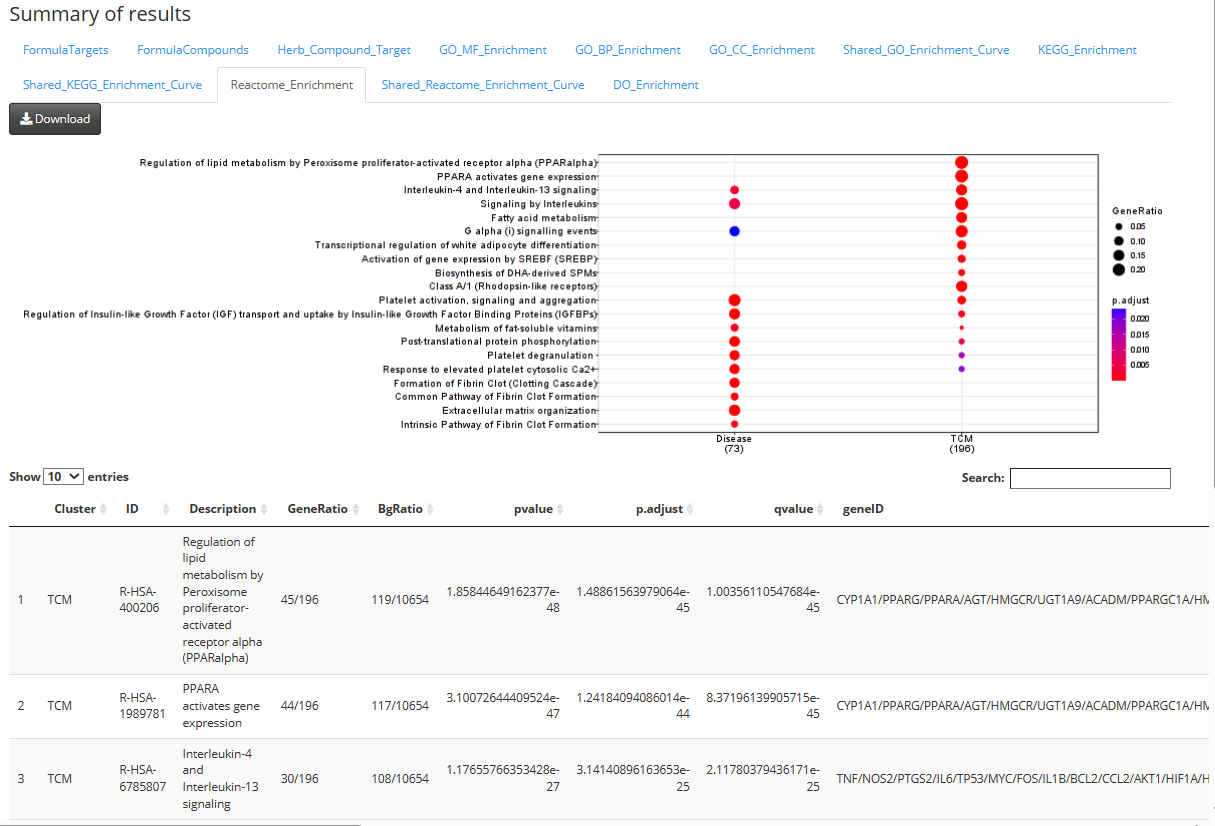

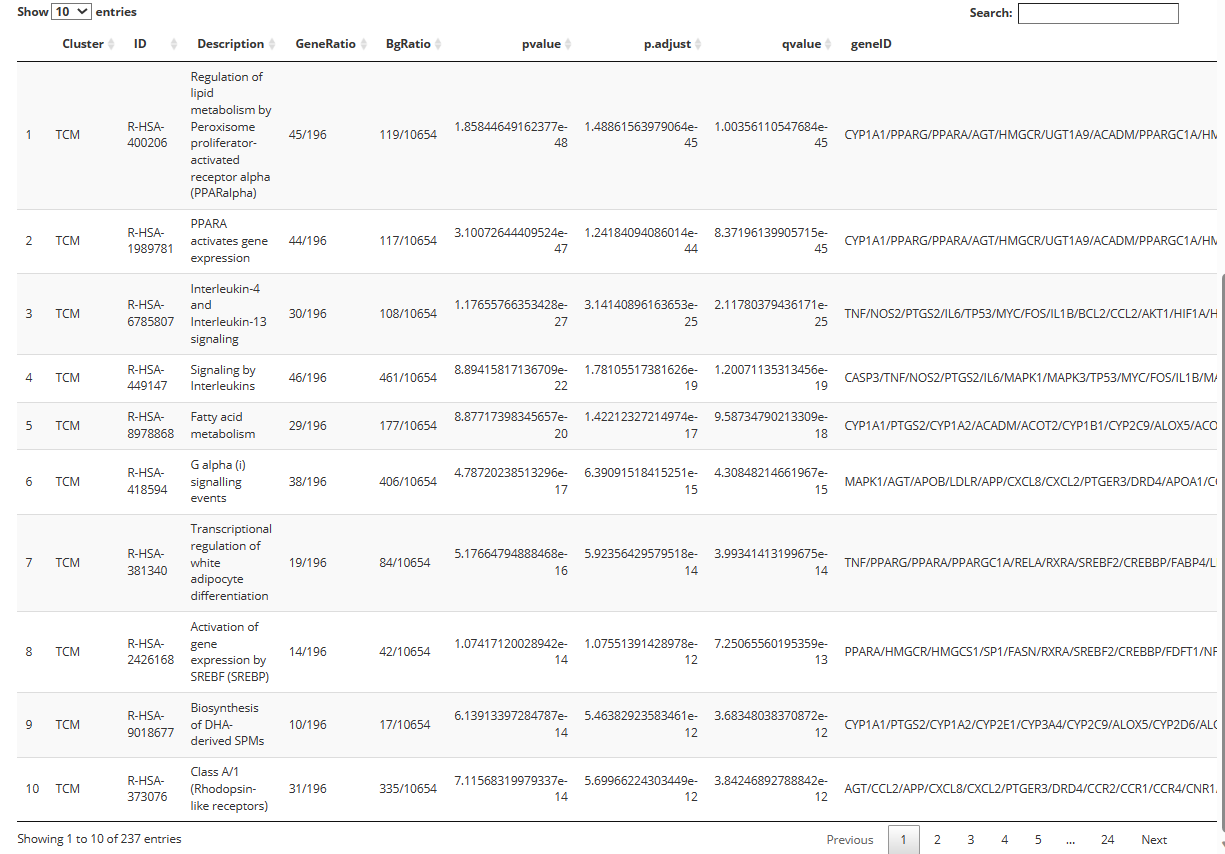


Figure S6 Formula Mechanism-Reactome Enrichment


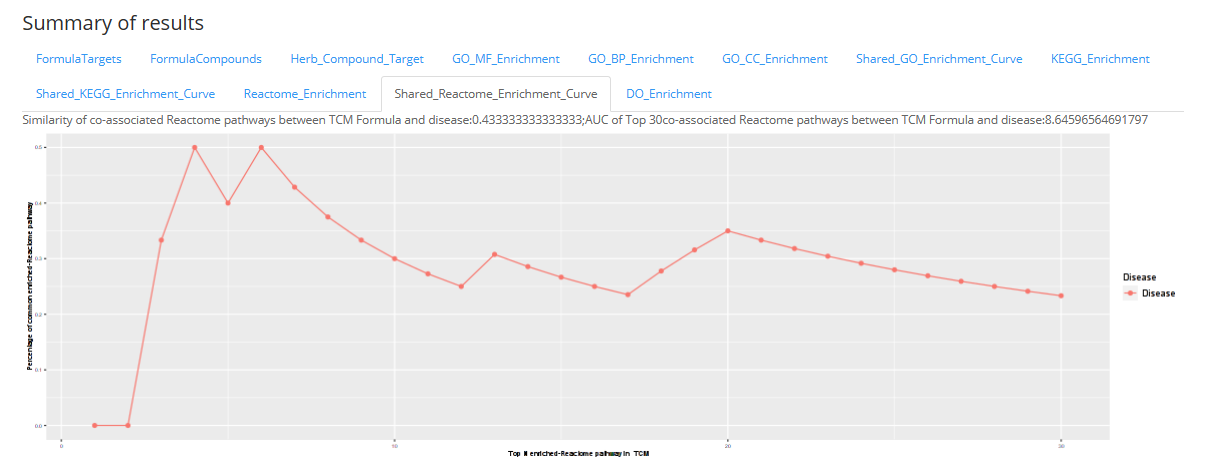


Figure S7 Formula Mechanism-Shared-Reactome-Enrichment-Curve


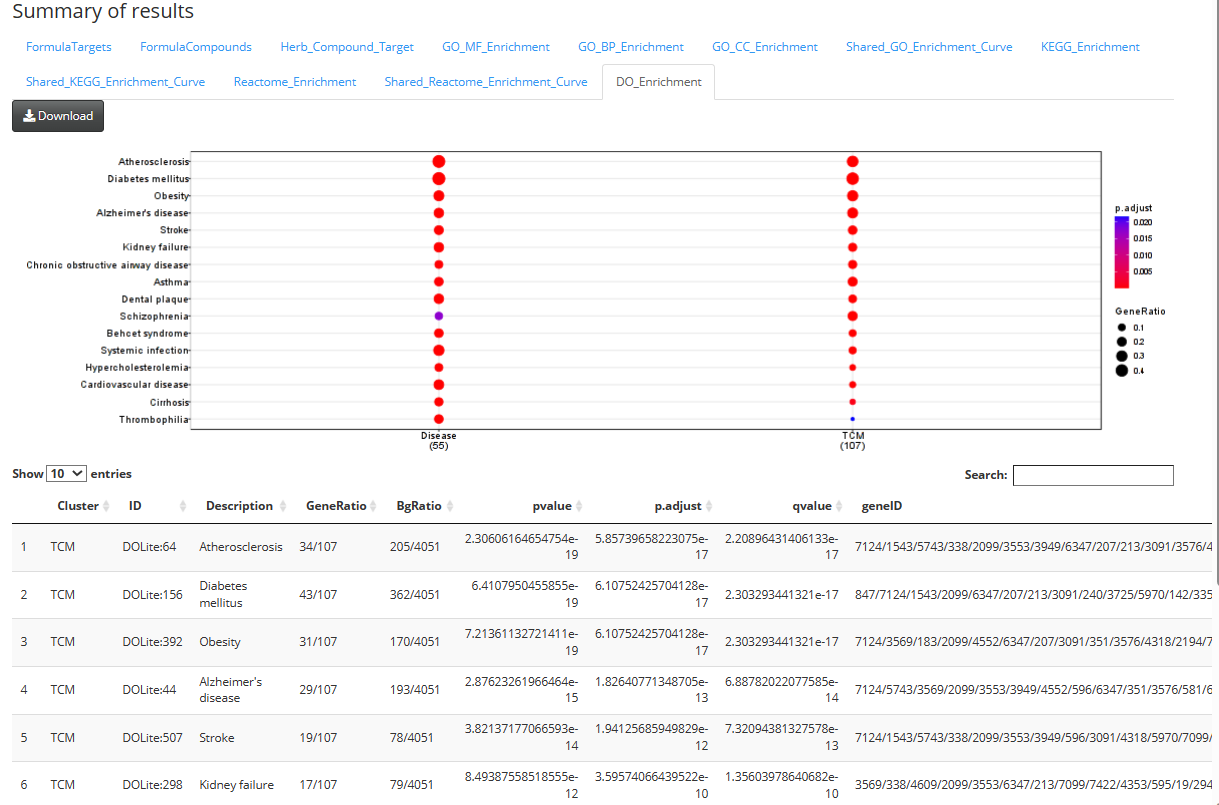

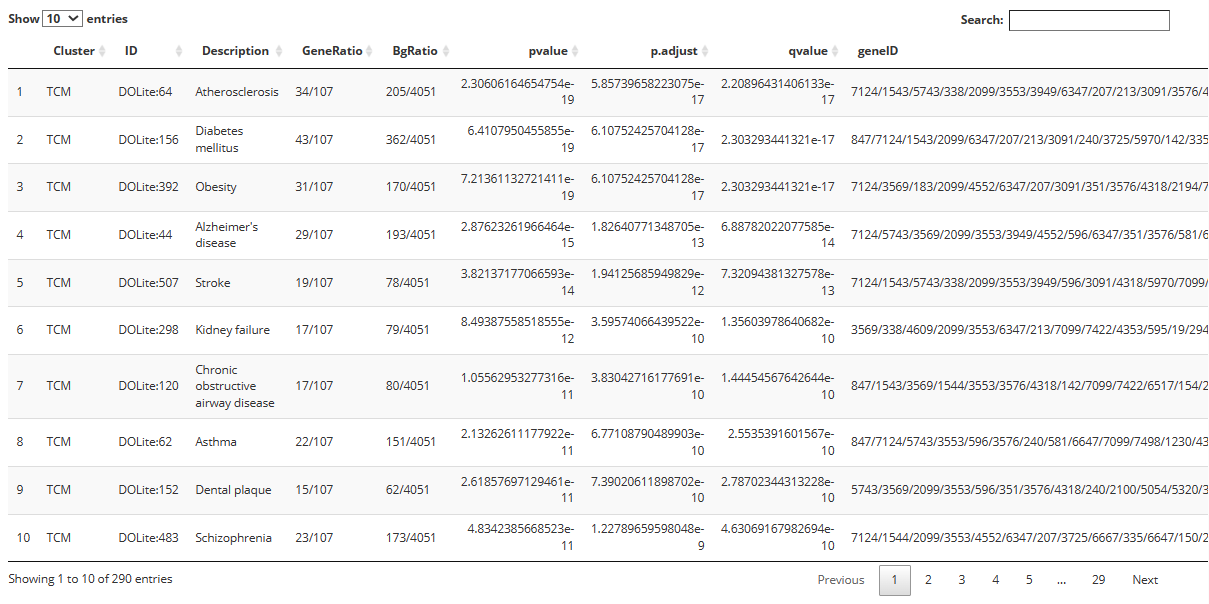


Figure S8 Formula Mechanism-DO Enrichment


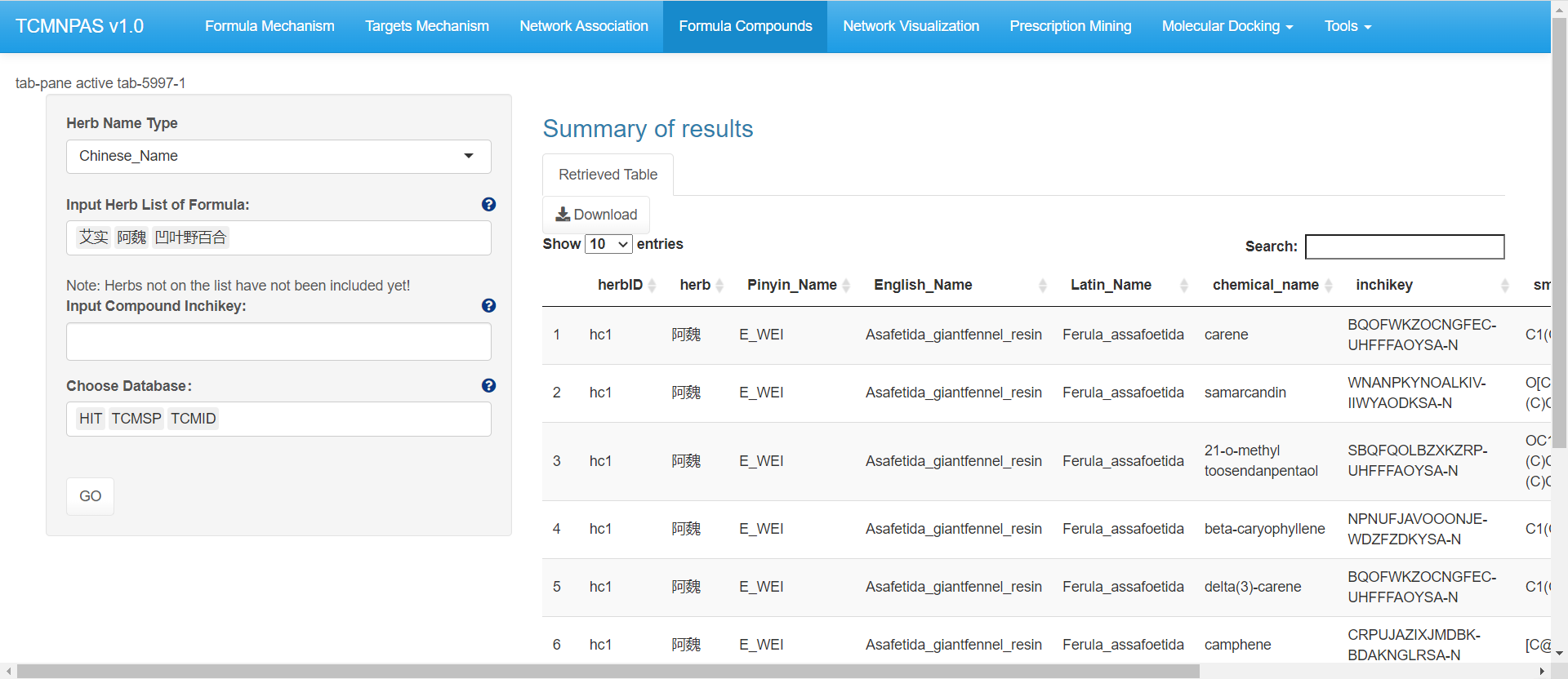


Figure S9 Formula Compounds


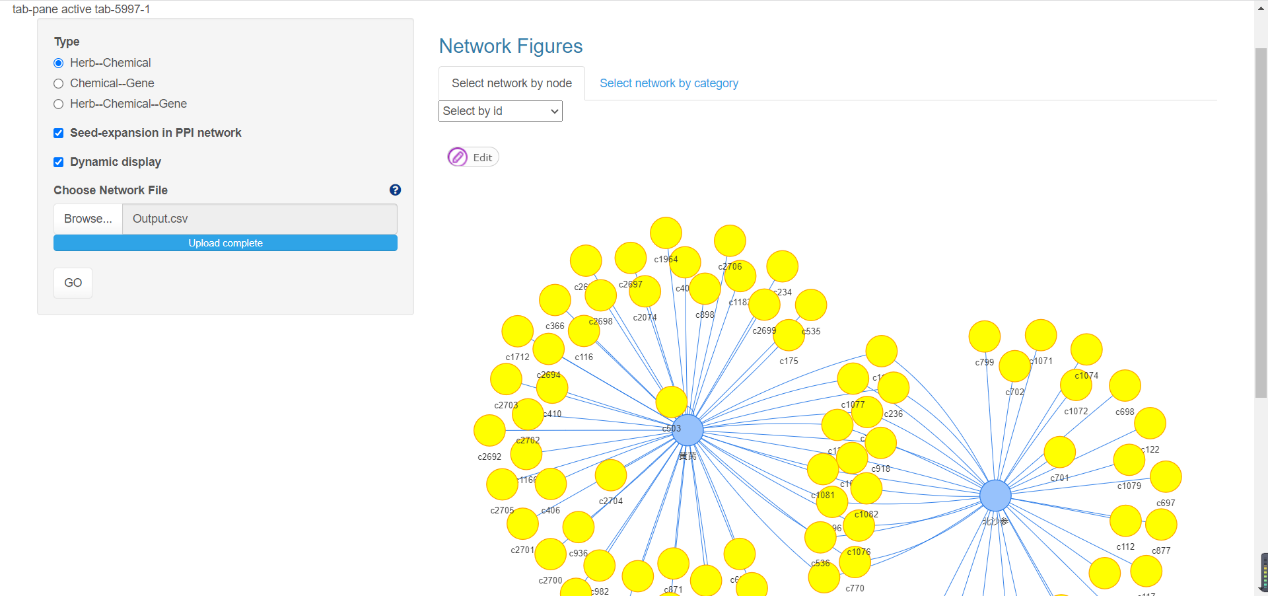


Figure S10 Network Visualization


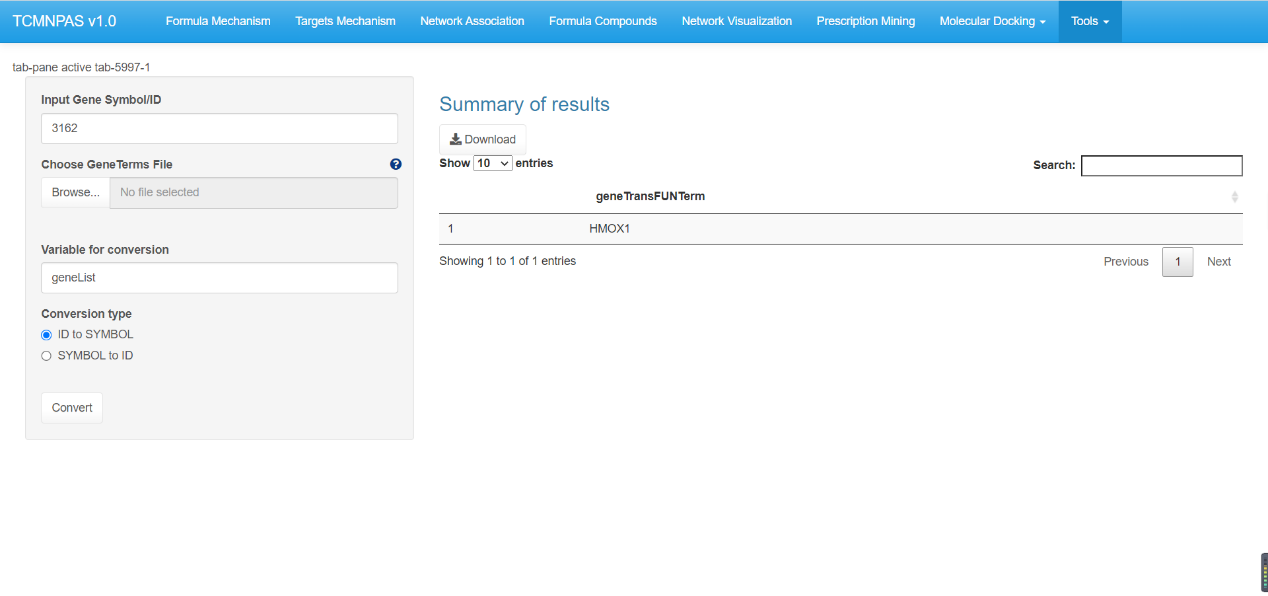


Figure S11 Tools-ID Conversion


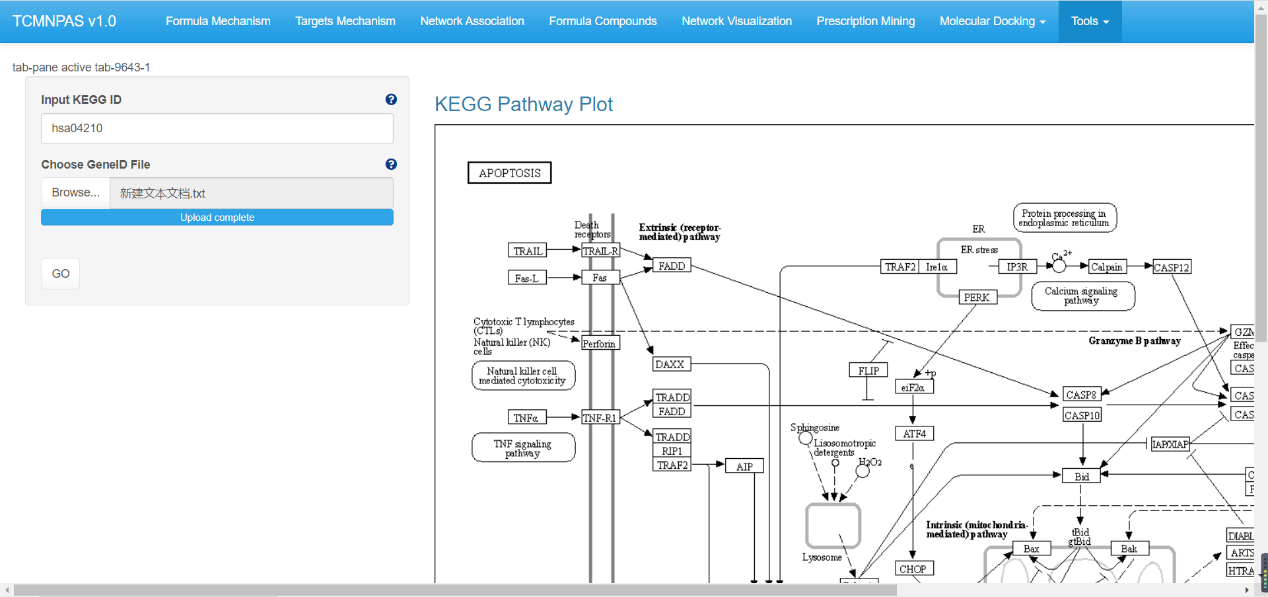


Figure S12 Tools-Seed in KEGG pathway


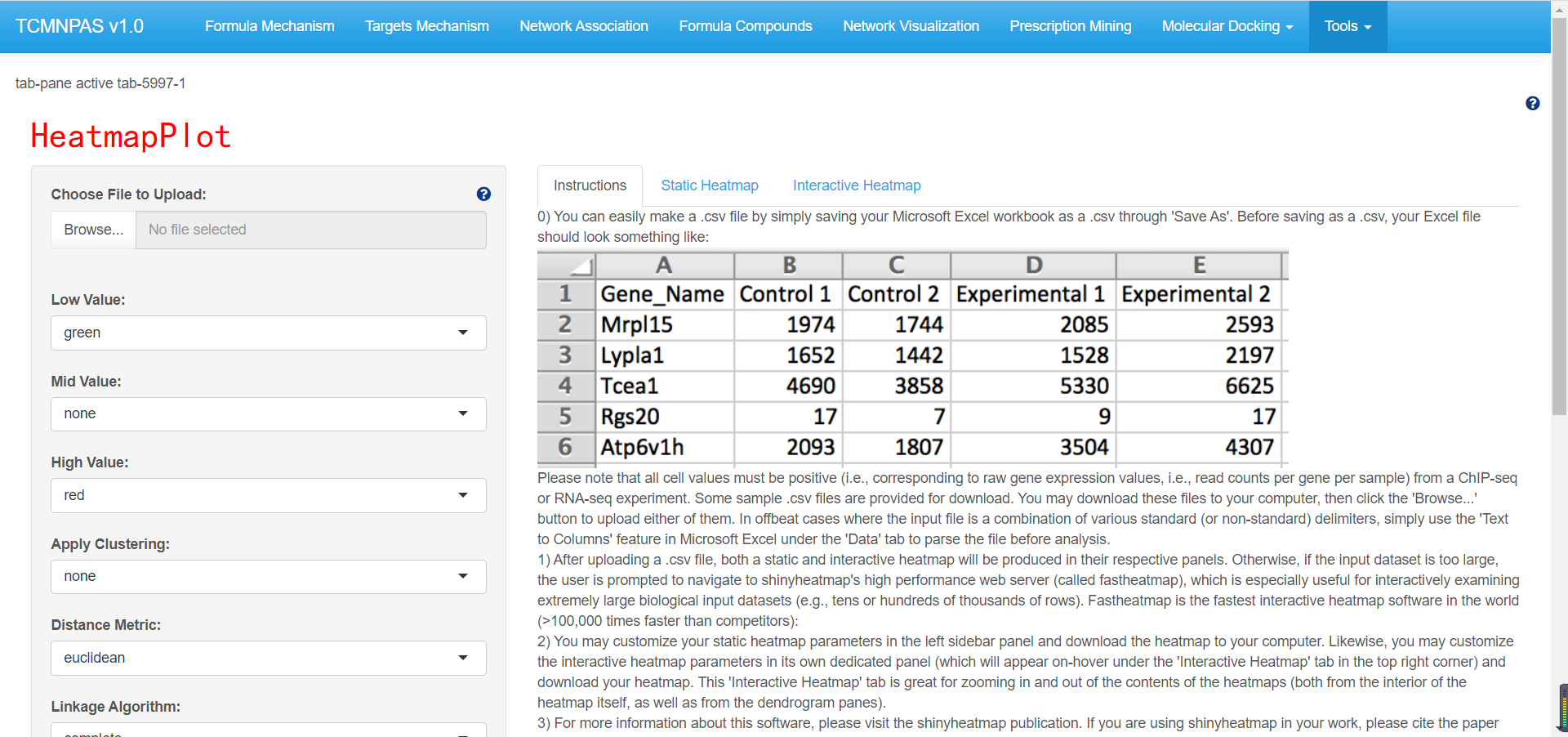


Figure S13 Tools-Heatmap


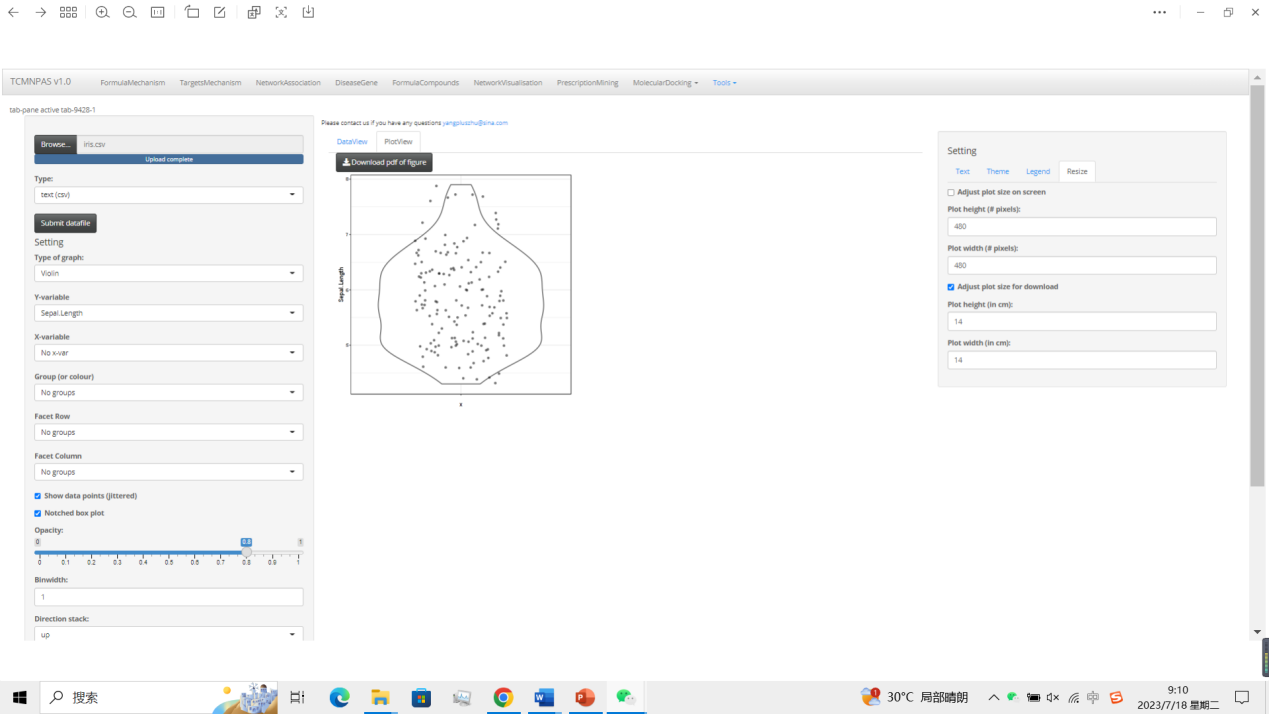


Figure S14 Tools-Data Visualization


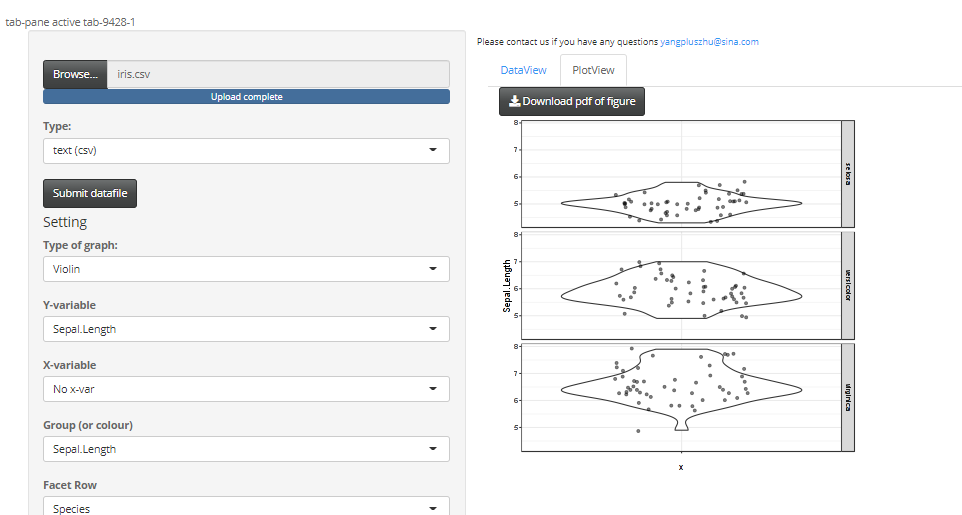


Figure S15 Tools-Data Visualization results

**References**

1. Zhang Y, Li X, Shi Y, Chen T, Xu Z, Wang P, et al. ETCM v2.0: An update with comprehensive resource and rich annotations for traditional Chinese medicine. Acta Pharm Sin B. 2023;13(6):2559-71.

2. Yan D, Zheng G, Wang C, Chen Z, Mao T, Gao J, et al. HIT 2.0: an enhanced platform for Herbal Ingredients' Targets. Nucleic Acids Res. 2022;50(D1):D1238-D43.

3. Wang P, Wang S, Chen H, Deng X, Zhang L, Xu H, et al. TCMIP v2.0 Powers the Identification of Chemical Constituents Available in Xinglou Chengqi Decoction and the Exploration of Pharmacological Mechanisms Acting on Stroke Complicated With Tanre Fushi Syndrome. Front Pharmacol. 2021;12:598200.

4. Kong X, Liu C, Zhang Z, Cheng M, Mei Z, Li X, et al. BATMAN-TCM 2.0: an enhanced integrative database for known and predicted interactions between traditional Chinese medicine ingredients and target proteins. Nucleic Acids Res. 2024;52(D1):D1110-D20.

5. Wu Y, Zhang F, Yang K, Fang S, Bu D, Li H, et al. SymMap: an integrative database of traditional Chinese medicine enhanced by symptom mapping. Nucleic Acids Res. 2019;47(D1):D1110-D7.

6. Fang S, Dong L, Liu L, Guo J, Zhao L, Zhang J, et al. HERB: a high-throughput experiment- and reference-guided database of traditional Chinese medicine. Nucleic Acids Res. 2021;49(D1):D1197-D206.

7. Tian S, Zhang J, Yuan S, Wang Q, Lv C, Wang J, et al. Exploring pharmacological active ingredients of traditional Chinese medicine by pharmacotranscriptomic map in ITCM. Brief Bioinform. 2023;24(2).

8. Zhang LX, Dong J, Wei H, Shi SH, Lu AP, Deng GM, et al. TCMSID: a simplified integrated database for drug discovery from traditional chinese medicine. J Cheminform. 2022;14(1):89.

9. Chen QF, Springer L, Gohlke BO, Goede A, Dunkel M, Abel R, et al. SuperTCM: A biocultural database combining biological pathways and historical linguistic data of Chinese Materia Medica for drug development. Biomed Pharmacother. 2021;144.
